# Excitotoxicity in amyotrophic lateral sclerosis: a key pathogenic mechanism

**DOI:** 10.1093/braincomms/fcag098

**Published:** 2026-03-20

**Authors:** Silvia Silva-Hucha, Rosendo G Hernández, Diego Baena-López, María Estrella Fernández de Sevilla, Carmen Paradas, Sara Morcuende

**Affiliations:** Departamento de Fisiología, Facultad de Biología, Universidad de Sevilla, Sevilla 41012, Spain; Instituto de Biomedicina de Sevilla, IBiS/Hospital Universitario Virgen del Rocío/CSIC/Universidad de Sevilla, Sevilla 41013, Spain; Centro de Investigación Biomédica en Red sobre Enfermedades Neurodegenerativas (CIBERNED), Madrid 28031, Spain; Departamento de Fisiología, Facultad de Biología, Universidad de Sevilla, Sevilla 41012, Spain; Departamento de Fisiología, Facultad de Biología, Universidad de Sevilla, Sevilla 41012, Spain; Departamento de Fisiología, Facultad de Biología, Universidad de Sevilla, Sevilla 41012, Spain; Instituto de Biomedicina de Sevilla, IBiS/Hospital Universitario Virgen del Rocío/CSIC/Universidad de Sevilla, Sevilla 41013, Spain; Centro de Investigación Biomédica en Red sobre Enfermedades Neurodegenerativas (CIBERNED), Madrid 28031, Spain; Departamento de Fisiología, Facultad de Biología, Universidad de Sevilla, Sevilla 41012, Spain

**Keywords:** neurodegenerative disorders, ALS, glutamate, motor neurons, glial cells

## Abstract

Amyotrophic lateral sclerosis is a complex neurodegenerative disease affecting motor neurons, characterized by the involvement of various factors, including oxidative stress, inflammatory processes, glutamate excitotoxicity, mitochondrial dysfunction, protein aggregation, axonal transport abnormalities, and apoptosis. The complexity of amyotrophic lateral sclerosis arises from its multifactorial aetiology involving diverse genetic, protein, metabolic, and cellular alterations. Mutations of different genes, such as *SOD1*, *C9ORF72*, *TARDBP*, and *FUS*, have been identified as critical contributors to disease pathophysiology through their facilitation of aberrant protein misfolding and aggregation. All these factors disrupt glutamate homeostasis, leading to calcium-mediated neurotoxicity. Under oxidative stress, motor neurons exhibit a diminished capacity to regulate calcium influx, along with impaired functioning of the mitochondria and endoplasmic reticulum, further compromising cellular integrity. Dysregulation of glutamate signalling also triggers astrocytic stress responses, leading to reduced glutamate clearance, thus worsening neuronal damage through excitotoxic mechanisms. These factors contribute to the excessive production of reactive oxygen species, which exacerbates glutamate imbalance and establishes a detrimental cycle of neuronal damage and glial dysfunction, ultimately intensifying excitotoxicity. This review aims to highlight the role of excitotoxicity in motor neuronal degeneration and to explore the molecular mechanisms underlying the pathogenesis of amyotrophic lateral sclerosis. It also examines current therapeutic approaches, including approved treatments and ongoing clinical trials to reduce excitotoxicity, while emphasizing the urgent need for novel, targeted strategies. Given the lack of definitive diagnostic tools and curative therapies, advancing our understanding of the molecular mechanisms driving excitotoxicity and neurodegeneration is, therefore, crucial for the development of more effective, disease-modifying treatments to slow amyotrophic lateral sclerosis progression.

## Teaser

In this study, Silva-Hucha *et al*. highlight excitotoxicity as a central mechanism in amyotrophic lateral sclerosis, driven by aberrant glutamate release and impaired glutamate clearance. It explores the roles of EAAT2 dysfunction, calcium dysregulation, oxidative stress, and organelle damage, underscoring the need for targeted therapies and better disease models.

## Graphical Abstract

**Graphical Abstract:**
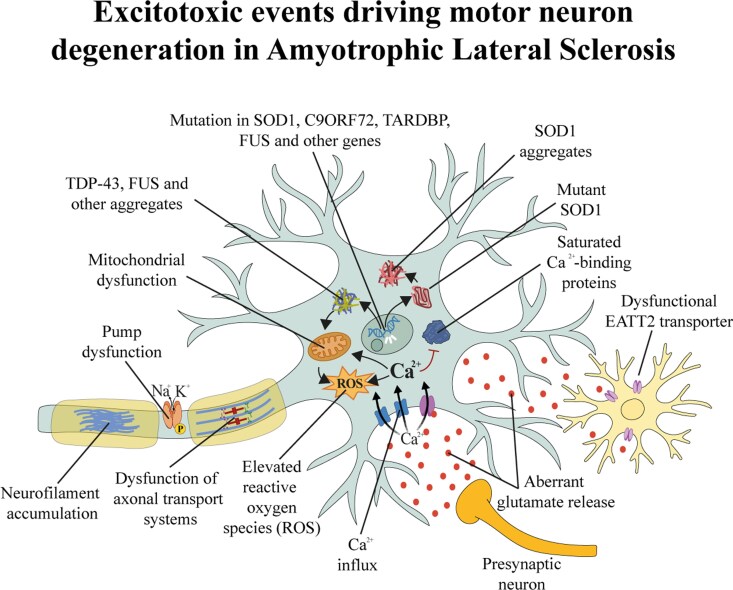

## Introduction

### Amyotrophic lateral sclerosis

Amyotrophic lateral sclerosis (ALS) is characterized as an adult neurodegenerative disease that causes progressive degeneration of motor neurons in the spinal cord, brainstem and cortex, triggering progressive atrophy of skeletal musculature and thus reduced voluntary movements, including limb, bulbar and respiratory movements.^1-3^ ALS affects 5 out of every 100 000 people worldwide, regardless of its origin.^4^ It occurs more frequently in men than in women, and the first symptoms of the disease are usually detected around the age of 50,^5,6^ increasing its frequency between 60 and 79 years of age.^7,8^

ALS presents as a combination of upper (UMN) and lower (LMN) motor neurons dysfunction affecting the bulbar, cervical, thoracic, and/or lumbar segments.^9^ ALS usually initiates in the spinal cord, with the first symptoms appearing in the limbs in 80% of cases or bulbar regions in the remaining 20%.^10^ The neuronal loss usually begins focally and spreads, with symptoms manifesting as subtle cramps, spasticity or fasciculations that progress to weakness in the limbs and bulbar muscles, ultimately leading to paralysis of nearly all skeletal muscles, respiratory involvement with the need for mechanical ventilation and feeding tube.^10-14^ Patients die as a result of respiratory failure 3–5 years after diagnosis.^15,16^ Besides, around 60% of ALS patients show frontotemporal deterioration, which is often accompanied by frontotemporal dementia (FTD) in 15% of cases.^17^ Depending on the predominant location of the primary pathology, eight recognized phenotypes of ALS have been identified,^18-20^ each presenting distinct clinical manifestations, as summarized in Table 1.

**Table 1:** Clinical phenotypes of ALS based on primary regions affected

Phenotype	Key features	Primary affected areas	Additional notes
Classic (Charcot’s)	Muscle weakness in upper/lower limbs; clear but not predominant pyramidal signs	Upper and lower motor neurons (UMN and LMN)	Most common phenotype; hallmark of ALS; progresses to involve other regions
Bulbar	Dysarthria and/or dysphagia, tongue atrophy and fasciculations; no spinal involvement in first 6 months	Bulbar LMN	Often associated with faster progression; may later involve spinal regions
Flail arm	Gradual, proximal weakness and atrophy in upper limbs	LMN (upper limbs)	Slower progression; may remain confined to upper limbs for extended time
Flail leg	Progressive, distal weakness and atrophy in lower limbs	LMN (lower limbs)	May mimic peripheral neuropathies; generally slower progression
Pyramidal	Predominantly spastic paraparesis or tetraparesis; hyperreflexia, Babinski/Hoffmann signs, pseudobulbar signs; also has LMN signs from onset	Predominant UMN, with LMN evidence	Strong UMN signs from early on; coexisting LMN signs essential for ALS diagnosis
Respiratory	Respiratory dysfunction (e.g. orthopnoea, dyspnoea) at onset; weakness starts in respiratory muscles	UMN and LMN (respiratory muscles)	Can be initially misdiagnosed as primary pulmonary disease
Pure LMN	Progressive weakness, atrophy, fasciculations; flaccid limbs; no significant UMN signs; consistent with progressive muscular atrophy	LMN only	Also termed progressive muscular atrophy (PMA); slower progression than typical ALS
Pure UMN	Spasticity, hyperreflexia, Babinski/Hoffmann signs, pseudobulbar affect; minimal atrophy; consistent with primary lateral sclerosis	UMN only	Also known as primary lateral sclerosis (PLS); may evolve into ALS with time

#### Sporadic and familial ALS

Approximately 90–95% of ALS cases are sporadic (sALS), while 5–10% of patients have an inherited nature and are known as familial ALS (fALS), due to the presence of a family history of ALS or sometimes related to other neurodegenerative diseases.^21^

Pathogenic variants in four genes, *SOD1*, *C9ORF72*, *TARDBP*, and *FUS*, have been established as prevalent genetic causes of fALS thus far.^22-24^ Collectively, mutations in these genes are estimated to account for approximately one-third of fALS cases.^19,25-27^ Variants in *SOD1*, *FUS*, and *TARDBP* have also been identified in approximately 1–2% of cases initially classified as sALS. One-fifth of the fALS cases carry mutations in the *SOD1* gene, but several studies have indicated that SOD1 dysfunction may also play a pathogenic role in sALS.^28^ To date, 177 known mutations in *SOD1* have been associated with the pathogenesis of ALS.^29,30^

The formation of protein aggregates, specifically of the TAR DNA-binding protein 43 (TDP-43), is the universal hallmark of the disease. This proteinopathy is present in ∼97% of cases of ALS, regardless of *TARDBP* gene mutations,^14,31^ and is also present in ∼50% of FTD cases.^17,32^ Mutation in *FUS* contributes to the formation of components of stress granules, such as p62 and TDP-43-positive aggregates.^33^ In addition, the majority of the heritable risk for FTD is attributable to autosomal dominant hexanucleotide repeat expansions in the *C9ORF72* gene, which are also implicated in the FTD-ALS disease spectrum and are characteristically associated with TDP-43 proteinopathy.^34,35^ These aggregates result from post-translational modifications of nuclear TDP-43 and are closely linked to neurodegenerative progression due to impaired axonal transport.^36^ It is hypothesized that compromised autophagy may play a role in the buildup of cytoplasmic aggregates.^37^

Additionally, recent findings have identified mutations in new genes involved in ALS, which affect the processes of cellular morphogenesis and membrane trafficking,^38^ autophagy and mitophagy,^30^ and regulation of inflammatory and neuroprotective responses.^39,40^ So, although most ALS cases are sporadic, a subset is familial and linked to mutations in genes which contribute to protein aggregation, particularly TDP-43 pathology, highlighting shared molecular mechanisms across ALS neurodegenerative disorder.

#### Pathophysiology aspects

The initial cause that triggers the selective motor neuron degeneration in patients with ALS remains unidentified. Although post-mortem human tissue analysis confirms that degeneration and death of motor neurons is the pathogenic basis of ALS,^41,42^ the disease is increasingly recognized as a disorder of the entire motor system. The degenerative process includes not only the UMN and LMNs, but also neuromuscular junctions, their innervated muscle fibres, and various paracrine cell types that support motor function.^12^ Emerging evidence suggests that it also involves early distal axonopathy and denervation at the neuromuscular junctions, particularly in limb muscles, reinforcing the concept of ALS as a motor system disease rather than a purely neuronal disorder.^43-47^ Current findings indicate that motor neuron degeneration in ALS arises from complex interactions involving multiple cell types and components of the motor system.^12,29^

**Figure 1:**
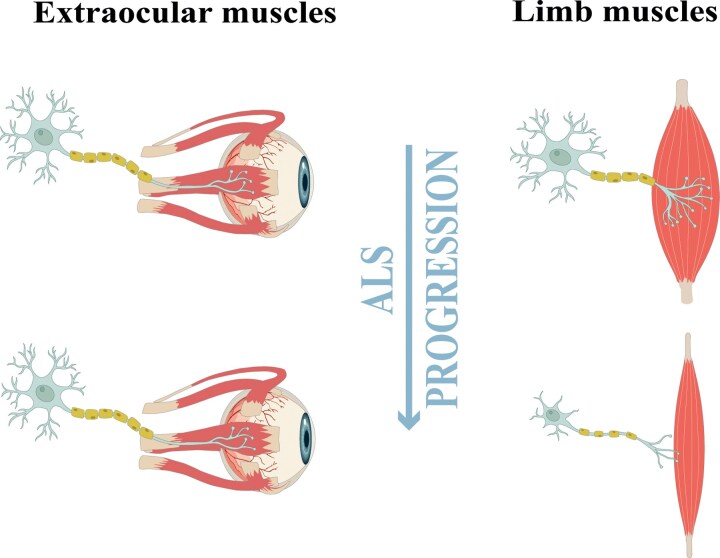
**ALS exhibits differential vulnerability among motor systems.** Motor neurons innervating limb muscles are highly vulnerable to degeneration, contributing to progressive motor decline. In contrast, motor neurons and muscle fibres of the oculomotor system are largely spared throughout the disease course, illustrating a distinct pattern of resistance within specific motor circuits, partly linked to differential expression of glutamate receptors and transporters, as well as the ability to buffer excess Ca^2+^ within motor neurons in response to oxidative stress.

It is remarkable that specific populations of motor neurons show greater resistance to degeneration and persist until the last stages of the disease, compared with other motor neurons that degenerate earlier (Fig. 1). The extraocular motor neurons, which innervate the extraocular muscles, remain unaffected in both human ALS patients and mouse models, in contrast to other more vulnerable motor neurons populations, such as those controlling the tongue, facial muscles and limbs. This differential vulnerability is partly driven by variations in the expression of glutamate receptors and transporters, which render specific motor neuron populations more susceptible to glutamate-mediated calcium overload and subsequent degeneration.^48-51^ Some proposed explanations for this differential survival involve mechanisms related to excitotoxicity, which will be discussed later.

Other studies determine that alterations in the skeletal muscle fibres trigger structural damage in motor neurons, causing these nerve cells to suffer damage to their structure even before neurodegenerative processes develop within the CNS as a result of damage to the muscle fibres.^44,45^ These findings support the hypothesis that skeletal muscle pathology may play an active role in ALS pathophysiology, potentially initiating or accelerating degenerative cascades in motor neurons.

Recently, a scientific study has provided the first evidence that a possible cause of fALS is the accumulation of junk proteins in motor neurons, proposing a new hypothesis for the disease. This study suggests that ALS has a similar origin to ribosomopathies, where an excessive accumulation of dysfunctional arginine-rich ribosomal proteins proves toxic, impairing cellular function and ultimately leading to motor neuron death.^52^

Among the various proposed factors, oxidative stress and protein aggregation are thought to be primarily responsible for the molecular pathogenesis triggered^53^ due to effects on neuronal viability, which is believed to depend on a delicate balance between survival and death signalling under both physiological and pathological conditions.^54,55^ This hypothesis supports the idea that the gain of excitatory signalling in motor neurons would increase calcium (Ca^2+^) influx and, therefore, increase the depolarization of motor neurons, which induces the development of excitotoxic processes.^56,57^ Excitotoxicity, driven by excessive activation of glutamate receptors, is not only a defining feature of ALS but also represents a central pathogenic mechanism in other neurodegenerative disorders, including Alzheimer’s, Parkinson’s, and Huntington’s disease. Although the overarching pathway involves Ca^2+^ overload, oxidative stress, mitochondrial dysfunction, and activation of cell death pathways, each condition exhibits distinct features in the way these processes are initiated, modulated, and amplified.^58^

In this review, we examine the multifactorial mechanism underlying the neurodegenerative processes triggered during ALS, with a primary focus on the pivotal role of excitotoxicity in the neurodegenerative processes. We explore how glutamate dysregulation and Ca^2+^-mediated toxicity serve as key drivers of motor neuron degeneration. Additionally, we consider the contribution of paracrine signalling and the specific roles of neighbouring cell types in modulating neuronal vulnerability and disease progression throughout the motor system.

### Role of excitotoxicity in neurodegeneration

Although the precise mechanisms underlying ALS remain incompletely understood, accumulating evidence suggests that excitotoxicity plays a significant role in its pathogenesis. Excitotoxicity is caused by high concentrations of glutamate in the synaptic space,^59^ as a consequence of an aberrant excess of glutamate release by neurons and a decrease of its reabsorption by astrocytes. Excitotoxicity temporal dynamics involve both acute and secondary processes. Acute excitotoxicity refers to rapid neuronal death triggered by excessive glutamate receptor activation. In contrast, secondary excitotoxicity represents a slower, progressive mechanism driven by chronic glutamate dysregulation and heightened motor neuron vulnerability due to low Ca^2+^-buffering capacity. This form is more consistent with the clinical and pathological features of ALS, where subtle but sustained elevations in extracellular glutamate contribute to cumulative neuronal stress and degeneration over time.^60^

Several studies using the transgenic ALS model (*Sod1*^G93A^) reinforce the evidence that there are alterations in presynaptic glutamate release. In those animals, even during presymptomatic stages, the glutamate release machinery appears to be excessively active.^61^ These early events in the pathogenesis of ALS contribute to excitotoxicity and increased vulnerability of motor neurons.^61-63^ This cascade leads to a persistent overstimulation of excitatory neurotransmitter receptors, in particular glutamate receptors, resulting in excessive influx of Ca^2+^ and sodium (Na^+^) ions into neurons that triggers neuronal degeneration and death.^64^

Other studies suggest that excitotoxicity in ALS is probably not only caused by ‘too much stimulation’, but also by neurons overreacting to stimuli that could be within a relatively normal range, i.e. due to changes in their internal biology.^65^ Therefore, dysregulation of glutamate homeostasis and impaired glutamate clearance contribute to increased synaptic excitability and motor neuronal degeneration, which are particularly vulnerable to this process.^66^ Understanding the mechanisms of excitotoxicity in ALS is crucial not only for elucidating disease progression but also for developing targeted therapeutic strategies aimed at preserving motor neuron function and slowing clinical decline. This phenomenon has been linked to both sporadic and familial forms of the disease.^67^

**Figure 2:**
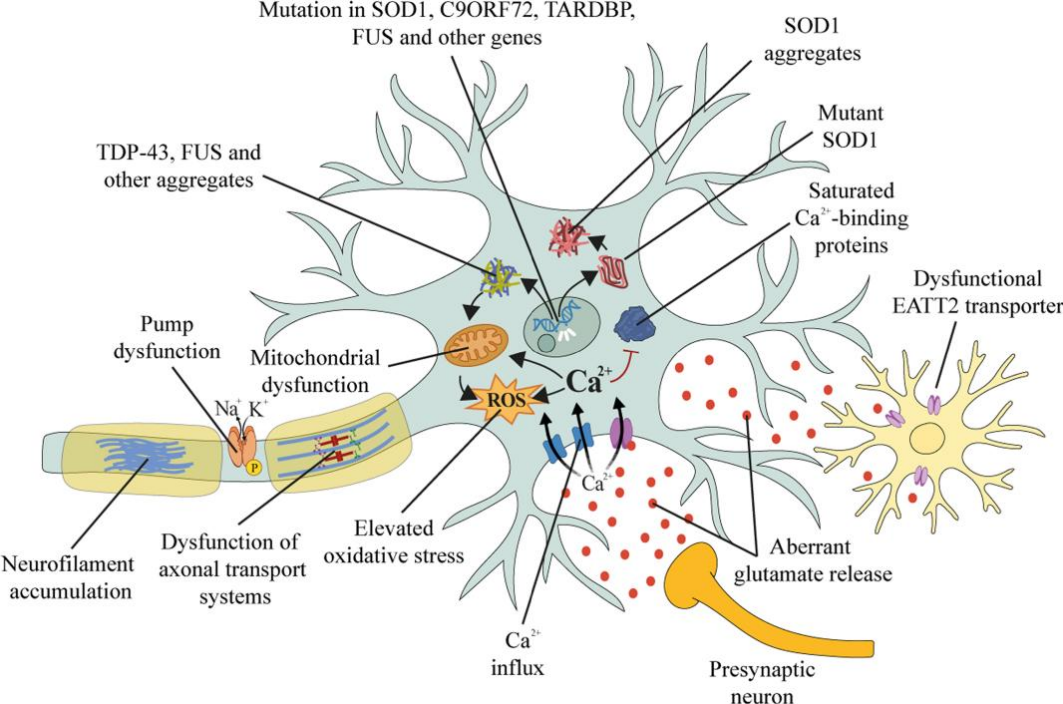
**Pathophysiological mechanisms underlying ALS.** ALS is characterized by a complex interplay of interconnected pathogenic processes. Aberrant glutamate release occurs both in the early and late stages of the disease, and glutamate-mediated excitotoxicity induces excessive Ca^2+^ influx, which motor neurons struggle to buffer, promoting neurodegeneration. Dysfunctional EATT2 transporters in astrocytes fail to clear glutamate from the extracellular space, and additionally, mutant glial cells secrete more glutamate, further contributing to motor neuron degeneration. The dysregulated Ca^2+^ homeostasis also contributes to the generation of ROS, exacerbating oxidative stress. Genetic mutations in *SOD1*, *C9ORF72*, *TARDBP*, and *FUS* disrupt RNA processing and promote toxic protein aggregates. In particular, mutant SOD1 enhances oxidative damage, impairs mitochondrial function, disrupts neurofilament organization, and compromises axonal transport, collectively undermining motor neuron viability.

This paper focuses on the role of excitotoxicity in the disease and how it is one of the main drivers of the associated neurodegenerative processes. To illustrate this, the chronological sequence of events and the key molecular mechanisms triggered by excitotoxicity are outlined and detailed below and are summarized in Fig. 2:

- Impaired glutamate clearance: Normally, glutamate released during neurotransmission is rapidly cleared from the synaptic cleft by astrocytes via excitatory amino acid transporter 2 (EAAT2, also known as GLT-1). In ALS, the function or expression of EAAT2 is reduced, leading to elevated extracellular glutamate levels.^66,68^
- Excessive glutamate release: As noted above, in the *Sod1*^G93A^ mouse, even before symptoms emerge, the mechanisms regulating glutamate release in spinal cord glutamatergic synapses appear to be overactive, resulting in excessive glutamate output and the triggering of excitotoxic cascades.^62^ In parallel, pathological astrocytes not only fail to clear glutamate but also actively release it through aberrant exocytosis.^69^ Activated microglia further elevate extrasynaptic glutamate by upregulating the cystine/glutamate antiporter (system Xc^−^).^69-71^
- Overactivation of glutamate receptors: Excess extracellular glutamate induces functional alterations of metabotropic and ionotropic receptors on motor neurons, specifically mGluR1 and mGluR5 metabotropic glutamate receptors,^72^ and *N*-methyl-D-aspartate (NMDA) and α-amino-3-hydroxy-5-isoxazole propionate (AMPA) ionotropic receptors,^73,74^ leading to prolonged channel opening and excessive Ca^2+^ influx into the cells. Additionally, dysfunction of the transduction mechanisms of mGluR1 and mGluR5 subunits of metabotropic glial cell receptors is also implicated in the development of the disease.^75^
- Intracellular Ca^2+^ overload: Motor neurons in ALS exhibit elevated expression of Ca^2+^-permeable AMPA receptors, reduced intracellular Ca^2+^-buffering proteins, and limited capacity to handle excessive Ca^2+^. These factors contribute to intracellular Ca^2+^ overload, which is highly toxic and promotes neuronal degeneration.^74,76^
- Oxidative stress and mitochondrial dysfunction: Elevated intracellular Ca^2+^ disrupts mitochondrial function, leading to the overproduction of reactive oxygen species (ROS). This oxidative stress damages essential cellular components, including DNA, proteins, and lipids.^77^
- Activation of cell death pathways: The combined effects of Ca^2+^ toxicity, oxidative stress, and mitochondrial dysfunction activate both apoptotic and necrotic pathways. In this situation, motor neurons begin to degenerate and die, contributing to the progressive muscle weakness and atrophy characteristic of ALS. The heightened vulnerability of motor neurons is attributed to their large size, high metabolic demand, low Ca^2+^-buffering capacity, reliance on astrocytic glutamate clearance, and the expression of Ca^2+^-permeable glutamate receptors.^78^

#### Impaired glutamate clearance: role of glial cells

Traditionally, glial cells have been considered to be no more than the structural support of the brain and spinal cord, in stark contrast to neurons, which have always been considered the key and fundamental building blocks of CNS function.^79^ For years, the motor decline in ALS was attributed exclusively to the loss of motor neurons in the brain and spinal cord. However, it is now well established that disease progression is significantly influenced by both the loss of function and the gain of toxicity of glial cells, which leads to the appearance of a reactive glial phenotype.^80^

These reactive glial cells take on both neuroprotective and neurotoxic functions during disease progression.^81,82^ The initial reactive response is thought to be neuroprotective, but over time, this reactivity becomes sustained and maladaptive, leading to chronic and toxic inflammation that contributes to the degeneration of surrounding motor neurons. This persistent inflammatory state disrupts CNS homeostasis, causing neuronal toxicity and cell death. Among the contributing factors, mutations in the *SOD1* gene within glial cells have been implicated in exacerbating these detrimental processes.^83^  *SOD1* mutation in glia is consistent with an inflammatory phenotype and a typical gliosis characterized by proliferative, hypertrophic and globular astrocytes.^84^ These results are supported by *in vivo* experiments in a transgenic mouse model, where the relevance of the specific deletion of the mutant *Sod1* gene in astrocytes and its direct involvement in the delay and/or substantial appearance of the progressive phase of the disease has been confirmed.^85-87^

One of the first mechanisms proposed to underlie ALS is glutamate excitotoxicity, as stated previously, which is the excessive activation of motor neurons that results from a failure of rapid synaptic glutamate clearance. Glutamate not only excites neurons but also depolarizes glial cells, specifically astrocytes.^88,89^ Under physiological conditions, astrocytes detect synaptically released glutamate by binding to ionotropic and metabotropic glutamate receptors and limit motor neuron activation through rapid glutamate retrieval. This function is mediated by the high-affinity EAAT2 transporter, which transports glutamate to the astrocyte and represents the most critical mechanism for glutamate clearance, playing a key role in regulating transmission and preventing excitotoxicity.^90^

Loss of EAAT2 has been observed in *Sod1* mutant rodent models and triggers a deficit in the rapid clearance of synaptic glutamate by astrocytes, resulting in a pathological process by glutamate excitotoxicity. Furthermore, it has been shown in ALS patients that EAAT2 transporters are decreased or defective.^66,91^ Many ALS patients have been found to have elevated glutamate levels in the CSF^92^ and a selective reduction of the astrocytic glutamate transporter EAAT2, further reinforcing the excitotoxic hypothesis of motor neuron degeneration.

Although astrocytic EAAT2 dysfunction has been widely implicated in ALS pathogenesis, some studies report that total EAAT2 protein levels in the CNS of *SOD1*^G93A^ mice remain unchanged,^93^ suggesting that regional or conformational alterations, rather than global downregulation, may underlie impaired glutamate clearance. This complexity is reflected in clinical trials targeting EAAT2 modulation: despite promising preclinical evidence that β-lactam antibiotics such as ceftriaxone upregulate EAAT2 and confer neuroprotection, a large multi-phase randomized trial failed to demonstrate therapeutic efficacy in ALS patients.^94,95^ These findings underscore the need to refine our understanding of EAAT2 regulation and localization in astrocytes, and to develop more targeted strategies for restoring glutamate homeostasis.

Additionally, it is well known that glial metabotropic glutamate receptors, specifically mGluR1 and mGluR5 subunits, modulate glutamate homeostasis, Ca^2+^ signalling, and astroglial inflammatory reactivity. In control murine models, mGluR5 activation can dynamically increase EAAT2 transporter function, helping to eliminate excess glutamate.^93^ However, in astrocytes with the *SOD1*^G93A^ mutation, mGluR5 signalling is altered, contributing to the loss of astrocyte glutamate clearance capacity and the improvement in glutamate uptake does not occur, favouring excitotoxicity.^96^ Furthermore, mGluR5 activation normally generates Ca^2+^ oscillations that appear to be necessary for modulating EAAT2. In *SOD1*^G93A^ astrocytes, the protein kinase C epsilon isoform (PKCε) is constitutively downregulated, altering the signalling pattern and preventing proper regulation of glutamate reuptake. Studies in which PKCε has been restored have shown that oscillations recover and uptake is regulated.^96^ As will be discussed in detail later, motor neurons are particularly vulnerable to glutamate-induced excitotoxicity due to their low expression of GluA2 subunits in AMPA receptors, which increases Ca^2+^ permeability and heightens their susceptibility to Ca^2+^-mediated toxicity.^97-100^ Astrocytes induce up-regulation of the GluA2 subunit of ionotropic receptors in co-cultured motor neurons,^101^ thus protecting them from excitotoxic damage. However, mutant SOD1 expression in astrocytes eliminates their GluA2 upregulation capacity and increases motor neuron vulnerability to AMPA receptor-mediated excitotoxicity.^101,102^ Therefore, impaired glutamate clearance by astrocytes, together with abnormal expression of GluA2 receptor subunits in motor neurons, may facilitate excitotoxic neuronal death in ALS. In addition, the reduced expression of the astrocytic inwardly rectifying potassium channel 4.1 (Kir4.1) observed in *Sod1* models may also contribute to motor neuron degeneration and hyperexcitability through impaired potassium homeostasis.^103,104^

Beyond glutamate clearance, astrocytes also convert glutamate into glutamine, which is recycled back to neurons for neurotransmitter synthesis. This glutamate–glutamine cycle is essential for maintaining excitatory and inhibitory balance in the CNS.^105^ Astrocytic dysfunction can disrupt this cycle, resulting in excessive extracellular glutamate and impaired GABA synthesis, both of which contribute to excitotoxicity and neuronal vulnerability.

These pathological processes collectively trigger astrogliosis, a process characterized by an increase in the number and size of glial cells, which often precedes neuronal damage. This response leads to the formation of reactive astrocytes, which actively contribute to neuronal degeneration and play an active role in mutation-mediated toxicity.^102^ Reactive astrocytes not only fail to protect neurons but actively contribute to their degeneration through dysregulated gliotransmission and the release of inflammatory mediators. The interplay between astrocytes and microglia further amplifies this excitotoxic environment, as reactive astrocytes promote neuroinflammation and lose their neuroprotective functions.^90,106^ It has been shown that proinflammatory mediators secreted by SOD1-reactive astrocytes promote microglial activation, meanwhile deletion of the *Sod1* gene in astrocytes leads to delayed microglia activation, highlighting a functional crosstalk between astrocytes and microglia.^12^ It is well known that microglial cells are activated in all types of ALS. The synthesis of mutant SOD1 by microglia is a critical determinant of rapid disease progression, as determined by selectively silencing the mutant *Sod1* gene in microglia^2^ or by using cell grafts to replace microglia expressing mutant SOD1 with normal microglia.^107^ In fact, in *Sod1* mutant mouse models, microglial cells show a neurotoxic phenotype and appear to contribute significantly to disease progression.^2,107^

Moreover, further evidence that these cells are involved in the neurodegenerative process is that blocking tumor necrosis factor alpha-induced microglial activation significantly prolongs the survival of transgenic ALS mice.^108^ However, the resulting microglial activation in both human and transgenic mouse models has been shown to trigger increased ROS production and the release of factors that can trigger the release of more glutamate from astrocytes and cause their oxidation.^109-111^ On top of that, classically activated neuroinflammatory microglia can induce a subtype of reactive astrocytes with neurotoxic properties.^112^ This subtype of astrocytes is present in several human neurodegenerative diseases, including ALS, being the activated microglia responsible for the neurotoxic phenotype displayed by ALS astrocytes. As microglial cells co-express potentially neurotoxic and neuroprotective factors during the disease,^113^ the role of astrocyte-microglia interaction in ALS has yet to be fully characterized.^114^

On the other hand, it has been shown that group I metabotropic glutamate receptors (mGluR1 and mGluR5) present in microglia also play a crucial role in regulating cell activation, the production of inflammatory mediators, the release of extracellular vesicles, and the modulation of the reactive phenotype of microglia. It has been demonstrated that the activation or blockade of these receptors in microglia can have dual effects (protective or detrimental), depending on the timing, intensity of the stimulus and the microenvironment, and can influence microglial activation and neuroinflammatory processes, both in normal conditions and in diseases such as ALS. In fact, in the case of traumatic brain injury, spinal cord damage, and other CNS pathologies, the expression of mGluR1/mGluR5 in microglia changes, and modulation of these receptors can alter the inflammatory response.^115^

In summary, while glial reactivity may initially serve a neuroprotective function, its chronic activation contributes significantly to the progression of ALS. Sustained glial activation leads to a pro-inflammatory environment that disrupts CNS homeostasis, exacerbates motor neuron vulnerability, and promotes neurodegeneration. Notably, mutations in genes such as *Sod1* within glial cells have been shown to intensify these toxic responses, negatively affecting the viability of motor neurons by producing elevated levels of extracellular glutamate, leading to increased motor neuronal neurodegeneration and progressive paralysis.

#### Excessive glutamate release

Excessive glutamate release represents a critical pathogenic mechanism contributing to excitotoxicity in ALS. Therefore, it is still controversial whether this represents a cause rather than an effect of the reduced glutamate clearance. Glutamatergic synaptosomes isolated from the lumbar spinal cord of *Sod1*^G93A^ ALS mice, that include several subpopulations of neurons, such as primary afferent neurons, projection neurons, specific interneurons and cortico-spinal upper motor neurons, exhibit abnormally elevated glutamate release. This phenomenon is mainly driven by the enlarged readily releasable vesicle pool and enhanced release facilitation, both of which reflect plastic changes in specific presynaptic mechanisms.^61-63^ Additionally, it has been shown that the activation of glycine transporters on glutamatergic terminals of transgenic animals causes increased glutamate release.^116^ This excessive glutamate output contributes directly to early excitotoxic stress, even before overt neurodegeneration becomes apparent. Rather than being a secondary consequence of neuronal injury, this heightened glutamate release reflects an intrinsic pathological alteration in motor neuron synaptic physiology that actively promotes excitotoxic vulnerability. However, spinal cord glutamatergic neurons are not the only cell types that release excessive glutamate into the extracellular space in this neurodegenerative disease, as glial cells also contribute to this pathological increase.

Astrocytes, which normally maintain extracellular glutamate homeostasis, undergo profound pathological changes during disease progression. Rather than solely losing their capacity for glutamate uptake, pathological astrocytes actively increase glutamate release through aberrant exocytosis,^69-71^ thereby amplifying excitatory stress on motor neurons. Astrocytes actively regulate extracellular glutamate through both vesicular and non-vesicular release mechanisms, which are normally balanced with glutamate uptake to maintain synaptic homeostasis.^117^ In ALS, this balance is disrupted: reactive astrocytes exhibit enhanced aberrant glutamate release, impaired Ca^2+^ signalling, and reduced transporter activity, contributing to excitotoxic stress on motor neurons. Mechanistic studies have shown that constitutive downregulation of PKCε in *SOD1*^G93A^ astrocytes disrupts mGluR5-mediated Ca^2+^ oscillations, impairing the activity-dependent modulation of glutamate uptake and thereby amplifying extracellular glutamate accumulation.^96^ This dysregulated glutamate release is not merely a consequence of neuronal damage but an active contributor to disease progression. Together, these findings indicate that pathological astrocytic glutamate release in ALS arises not only from overactive release mechanisms but also from uncoupled signalling pathways that normally coordinate release and uptake, highlighting the mGluR5–PKCε axis as a critical node in astrocyte-driven excitotoxicity. Targeting these astrocytic signalling pathways may therefore represent a promising therapeutic strategy to restore glutamate homeostasis and protect motor neurons in ALS.

Microglia also play a significant role in elevating extracellular glutamate levels. It is known that activated microglia contribute to glutamate-mediated excitotoxicity through non-vesicular release mechanisms, primarily via the cystine/glutamate antiporter system Xc^−^.^118^ Unlike neurons and astrocytes, microglia lack vesicular glutamate transporters and do not release glutamate through classical exocytosis. Instead, under inflammatory conditions, activated microglia upregulate system Xc^−^, exporting intracellular glutamate in exchange for cystine.^119,120^ This release contributes to extrasynaptic glutamate accumulation, which can overstimulate NMDA receptors on neurons and lead to Ca^2+^ influx and excitotoxic neuronal damage. This process is not incidental but a regulated response to neuroinflammation, amplifying excitotoxic cascades in ALS, complementing astrocytic dysfunction and contributing to a toxic microenvironment that accelerates motor neuron degeneration.

In summary, the increase in glutamate release from motor neurons, astrocytes, and microglia creates a toxic excitatory environment that accelerates motor neuron degeneration in ALS.

#### Overactivation of glutamate receptors: effects on motor neurons

Ca^2+^ serves as a vital intracellular signalling molecule that contributes to a wide range of physiological processes. For neuronal stability, cytosolic Ca^2+^ is kept at low level (around 100 nM) by regulating its entry and exit, bind it with Ca^2+^-buffering proteins, sequester it in intracellular compartments or removing it via Ca^2+^-ATPases. When excessive Ca^2+^ flows into the cell (Fig. 2), these buffering mechanisms become overwhelmed, leading to a sharp rise in cytoplasmic Ca^2+^ that triggers normally low-activity enzymes, resulting in the production of harmful ROS, mitochondrial dysfunction, disrupted energy metabolism, membrane depolarization, and ultimately apoptotic cell death.^97,99,100,121-124^ As the main excitatory neurotransmitter in the mammalian CNS, glutamate regulates many aspects of normal brain function such as cognition, learning and memory, synaptogenesis, synaptic plasticity, migration, cell differentiation, and long-term potentiation.^98,125-128^ One of the potential mechanisms of ALS pathogenesis is excitotoxicity caused by high concentrations of glutamate in the synaptic space,^59^ as a consequence of an excess of glutamate released by neurons and a decrease of its reuptake from the synaptic space by astrocytes. When glutamate is released from the presynaptic glutamatergic terminal, it binds to its high-affinity receptors located on the postsynaptic membrane of neurons and glial cells. However, an excess in synaptic glutamate transmission leads to an overactivation of the different types of glutamate receptors, which causes a massive influx of Ca^2+^ and Na^+^ ions into neurons and triggers the uncontrolled activation of harmful processes that eventually lead to membrane destruction, neurodegeneration, and cell death.^97,99,100,121-124^ It is known that these Ca^2+^-dependent toxic processes may play a pivotal role in the pathogenesis of ALS.^129^

**Figure 3:**
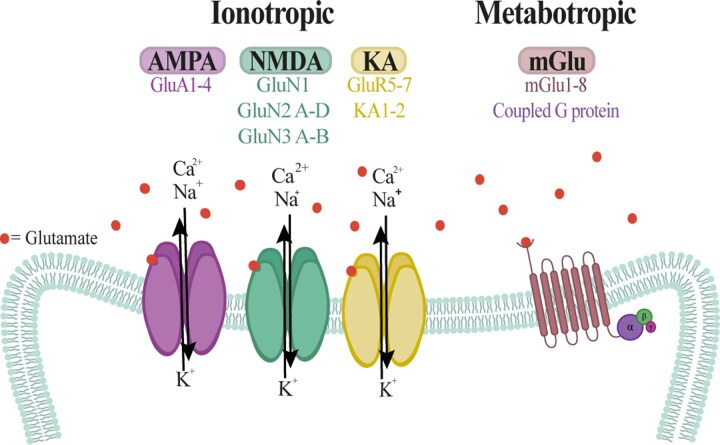
**Classification and schematic representation of glutamatergic receptors.** Glutamatergic receptors are classified into two main types based on their signalling mechanisms: ionotropic receptors, which function as ligand-gated ion channels, and metabotropic receptors, which signal through G protein-coupled pathways. AMPA, α-amino-3-hydroxy-5-methyl-4-isoxazolepropionic acid receptor; NMDA, *N*-methyl-D-aspartate receptor; KA, kainate receptor; GluA, glutamate receptor subunit A (AMPA-type subunit); GluN, glutamate receptor subunit N (NMDA-type subunit); GluR, glutamate receptor; mGlu, metabotropic glutamate receptor.

Two broad categories of glutamate receptors are known.^98^ The first category comprises the ionotropic receptors, which are ligand-activated ion channels, and includes the NMDA, AMPA and kainate receptors. The second category consists of the metabotropic G-protein-associated receptors (mGluR), which are coupled to the activation of intracellular secondary messenger^130,131^ (Fig. 3). Of the different types of glutamate receptors, the NMDA type is typically Ca^2+^ permeable and when overactivated can cause neuronal death in several brain areas where it is abundant, such as the hippocampus.^132-134^ However, in motor neurons, Ca^2+^ influx probably does not occur through NMDA receptors but rather through AMPA receptors, which mediate fast excitatory synaptic neurotransmission.^131^

Ionotropic AMPA receptors are heteromeric complexes composed of four subunits, GluA1–GluA4, with different combinations, which are encoded by separate genes and are abundantly expressed throughout the CNS.^131^ The presence of the GluA2 subunit is known to decrease the permeability of these receptors to Ca^2+^. Other authors consider that the role of the GluA2 subunit in Ca^2+^ permeability depends not only on its presence but also on the post-transcriptional RNA editing of the Q/R site present in its second membrane domain.^135^ Ultimately, changes in expression, or a deficit in post-transcriptional editing of GluA2, would be expected to have significant pathophysiological consequences by allowing Ca^2+^ entry into neurons through activation of endogenous glutamate.^98^ In a few words, glutamate receptors located in motor neurons have a relatively small amount of GluA2 subunit and, therefore, hyperactivation of AMPA receptors can lead to a massive influx of Ca^2+^ into the cell, triggering the activation of phospholipases, proteases, and endonucleases, which can induce apoptotic or necrotic cell death, ROS production and impaired mitochondrial function, with the consequent disruption of energy metabolism.^97-100^ All this could lead to motor neuron degeneration, suggesting that the absence of the GluA2 subunit in AMPA receptors is a critical factor in the selective vulnerability of motor neurons to excitotoxicity. Indeed, the removal of this subunit aggravates motor neuron degeneration in *Sod1^G93A^* mouse models.^136^

An experimental study found that the mRNA expression of GluA2, GluA3 and GluA4 is equivalent between resistant motor neurons and those vulnerable to degeneration in ALS.^137,138^ However, the authors postulate that in vulnerable motor neurons, there is a reduction in the transcription of GluA2 or other modifications in the subunit, which would end up affecting Ca^2+^ permeability and triggering the production of ROS during the progression of ALS, without therefore affecting GluA2 mRNA or protein levels.^137^

Other studies argue that AMPA receptor antagonists slow the progression of motor neuron degeneration in *Sod1^G93A^* mice.^139^ Furthermore, a recent *in vivo* model of acute spinal neurodegeneration has shown that the blockade of Ca^2+^-permeable AMPA receptors and the chelation of intracellular Ca^2+^ prevents motor neuron death, which is in line with one of the leading hypotheses on neuronal death in ALS,^98^ indicating that an excessive Ca^2+^ influx is responsible for motor neuron death. Therefore, the massive Ca^2+^ influx entering through AMPA-permeable receptors accumulates within mitochondria, possibly leading to energy deficits and the induction of apoptotic processes.^99,140^ Additionally, the use of blockers of cellular Ca^2+^ entry has been shown to provide neuroprotection, i.e. reductions in voltage-dependent Ca^2+^ and neurotransmitter entry show beneficial effects.^141,142^

On the other hand, metabotropic glutamate receptors (especially group I) have been shown to play a significant role in the pathophysiology of ALS too, acting at the intersection of excitotoxicity, synaptic regulation, and cell signalling.^143^ The metabotropic receptors mGluR1 and mGluR5 are Gq protein-coupled receptors that activate the phospholipase C pathway, which is involved in the production of inositol triphosphate and diacylglycerol, elevating intracellular Ca^2+^ and modulating multiple cellular cascades.^115^ Using the *SOD1*^G93A^ mouse model of ALS, it has been shown that group I metabotropic receptors exhibit increased sensitivity, i.e. low-intensity activations, trigger glutamate release in mutant mice.^144^ Thus, group I metabotropic glutamate receptors, specifically mGluR1 and mGluR5, show a functional increase in the regulation of glutamate release not only in advanced stages, but also in early stages of ALS progression.^72^ Likewise, a reduction in the constitutive expression of mGluR1 and mGluR5 in the *SOD1*^G93A^ mouse model of ALS has been shown to have protective effects, improving survival, delaying the onset of symptoms, slowing progression, and reducing neural damage.^145-147^ Therefore, negative modulators of mGluR1 and mGluR5 receptors may be promising therapeutic candidates in ALS.

In summary, the heightened vulnerability of motor neurons to degeneration has been linked to the high levels of intracellular Ca^2+^ due to their high permeability through glutamate receptors, via voltage-gated Ca^2+^ channels and to their limited endogenous Ca^2+^ buffering capacity, as will be discussed in the following section.

#### Intracellular Ca^2+^ overload: Ca^2+^ buffer proteins

The maintenance of intracellular Ca^2+^ homeostasis is vital for neuronal survival. This balance is regulated through a complex interplay of mechanisms, including Ca^2+^ uptake from the extracellular space via glutamate-activated channels and voltage-dependent Ca^2+^ channels, as well as Ca^2+^ uptake and release by intracellular organelles across their membranes. Additionally, intracellular buffering systems, such as the presence of Ca^2+^-binding proteins, modulate the intracellular concentration of Ca^2+^ ions, thereby safeguarding cellular metabolism and signalling pathways.^148^

Cytosolic Ca^2+^-binding proteins bind Ca^2+^ with high affinity and prevent large fluctuations in intracellular Ca^2+^ concentration (Fig. 2). The main Ca^2+^-binding proteins are parvalbumin (PV), calretinin (CR) and calbindin D-28k (CaBP-28K). These proteins are excellent markers of specific neuronal populations in various brain regions, including the neocortex, hippocampal formation, visual areas, cerebellum, and extraocular motor neurons.^149-151^ Overexpression of these cytosolic Ca^2+^ binding proteins appears to confer greater protection to motor neurons.^152-154^ Therefore, a differential distribution of Ca^2+^ buffering proteins could be responsible for the selective vulnerability observed among brainstem motor nuclei^50^ (Fig. 1).

Motor neurons are particularly vulnerable to intracellular Ca^2+^ overload due to their low Ca^2+^ buffering capacity.^155^ It has recently been demonstrated that cultured motor neurons exhibit insufficient mitochondrial capacity to buffer high intracellular Ca^2+^ concentrations after repeated activation of the AMPA receptor by kainate exposure.^156^ This suggests that their vulnerability to AMPA receptor-mediated excitotoxicity may be due to a deficient mechanism in intracellular Ca^2+^ homeostasis, limiting their ability to cope with sustained activation of Ca^2+^-permeable AMPA receptors, which are abundant in spinal motor neurons.^98^

In fact, it has been described that the more resistant motor neurons, which degenerate in later stages of the disease, such as those forming the oculomotor, trochlear, abducens, and Onuf’s nuclei, express significantly higher levels of CaBP-28k and PV proteins^152,157^ (Fig. 1). In contrast, these Ca^2+^-binding proteins appear to be absent or present in small quantities in the more vulnerable motor neuron populations that undergo early degeneration during the disease, such as cortical, spinal, and lower cranial nerve motor neurons.^157-160^ Additionally, a line of transgenic mice overexpressing PV has shown that increased PV expression can rescue vulnerable motor neurons from immune-mediated increases in intracellular Ca^2+^.^153,161^ Furthermore, cultured motor neurons from mice overexpressing PV are less susceptible to Ca^2+^-dependent kainate-induced excitotoxicity.^154^ Considering these results, it can be confirmed that PV may have a neuroprotective effect on motor neurons in various models of motor neuron degeneration.

However, other studies indicate that some Ca^2+^-buffering proteins, such as CaBP-28k, are absent in both vulnerable and resistant and that spinal motor neurons also express PV and CR.^155,162^ Additionally, in a murine model of epilepsy, the presence of CaBP-28k, PV or CR did not confer any resistance to neurodegeneration,^163^ suggesting that these Ca^2+^-binding proteins may not be reliable markers of resistance to degeneration in ALS. It is worth noting, however, that this study was conducted in the hippocampus and not in motor neurons.

All these studies and results have led to the hypothesis that differential expression of Ca^2+^-buffering proteins between resistant and vulnerable motor neurons contributes to non-uniform motor neuron degeneration in ALS.^157,159,164^ In fact, the buffering capacity of murine extraocular motor neurons is five to six times greater than that of hypoglossal or spinal motor neurons.^158,165,166^ However, it is still unclear which protein within this large family of Ca^2+^-buffering proteins underlies this enhanced buffering capacity. The results described here suggest that while PV may contribute to the greater buffering capacity of extraocular motor neurons compared with hypoglossal motor neurons, it may be less critical in establishing the greater buffering capacity of the extraocular relative to spinal motor neurons. Similarly, CR may impact the buffering capacity of spinal motor neurons, but it may be absent in all cranial motor neurons, including resistant motor neurons,^167^ indicating that it is unlikely to contribute to the differential resistance of motor neurons to degeneration. Instead, CaBP-28K has a high capacity for intracellular Ca^2+^ storage and is present in subsets of neurons that are resistant to neurodegeneration throughout the brain.^149,168-170^

Therefore, these differences in buffering capacity provide a plausible cellular explanation for the selective vulnerability and resistance exhibited by specific populations of motor neurons.

#### Oxidative stress: mitochondria and endoplasmic reticulum dysfunction in neurodegeneration

As has already been explained previously, among the numerous alterations in neuronal biology present in ALS, there are two common characteristics of the disease, which are the loss of Ca^2+^ homeostasis and deregulations in lipid metabolism. Given that both mitochondria and the ER are essential in these functions, the evidence suggests that alterations in cellular lipid metabolism and bioenergetics play a critical role during ALS processes.^171,172^ Lipids are the main components of cellular membranes and play essential roles in various cellular processes, such as energy storage, signalling, and membrane structure and fluidity. Dysregulation of lipid metabolism has been implicated in several neurodegenerative diseases, including ALS.^173^

Mitochondria have a role in controlling Ca^2+^ homeostasis in motor neurons to buffer and regulate ROS-dependent excitability.^174^ Impaired mitochondrial respiration has been widely reported to induce the degeneration of motor neurons by glutamate stimulation and environmental toxins.^175,176^ Motor neurons possess many Ca^2+^/K^+^ channels that, when activated, cause a rapid Ca^2+^ influx that, due to weak cytosolic Ca^2+^ buffering capacity, results in mitochondrial Ca^2+^ overload and strong ROS generation (Fig. 2). These features could largely explain the differential vulnerability to oxidative damage and mitochondrial abnormalities observed in different motor neuron populations during ALS.^177^ Indeed, the presence of different mitochondrial genetic alterations can significantly disrupt cell function, leading to impaired electron transport chain activity, increased activity of free radical scavenging enzymes, altered Ca^2+^ homeostasis and structural abnormalities within mitochondria. These dysfunctions suggest that mutations in mitochondrial DNA may play a pathological role in some forms of ALS, positioning mitochondria as a potential actor in the ALS pathological mechanism.^178^ This hypothesis is supported by multiple studies demonstrating that mitochondrial dysfunction is closely associated with elevated ROS production.^174^ It is well known that some observed phenomena, such as aggregate formation, neurofilament disruption and induction of apoptotic pathways (Fig. 2), could be a consequence of ROS generation or Ca^2+^ increase.^174,179^ Other studies confirm that increased ROS production by mitochondria in motor neurons is the result of mitochondrial Ca^2+^ overload following excitotoxic stimulation of AMPA/kainate receptors.^77^

ROS become highly toxic when their production surpasses the capacity of cellular antioxidant defences, such as SOD1, to neutralize them. This imbalance is particularly evident in patients with fALS linked to mutations in the *SOD1* gene. One contributing factor is the accumulation of mutant SOD1 proteins within or on the surface of mitochondria,^180^ which disrupts mitochondrial function. Additionally, these mutant proteins can interfere with key components of mitochondrial physiology, such as RNA molecules and molecular chaperones, further impairing mitochondrial integrity and contributing to neuronal degeneration.^181^ Simultaneously, the antioxidant function of SOD1 is compromised, and its aberrant interactions with various cellular molecules and organelles further contribute to elevated ROS levels.^182^ This chaos is thought to be due, in addition, to the multiple alterations caused by oxidative stress in RNA processing, such as the delocalization of TDP-43 and FUS proteins in patients, resulting in a mismatch in the metabolism of genetic material.^14^

Among the most critical ROS are peroxyl radicals (ROO), NO, superoxide radical anion (O^2−^), hydroxyl radical (OH) and some other non-radical species such as peroxynitrite (ONOO), simple oxygen (O_2)_ and hydrogen peroxide (H_2_O_2_).^183^ ROS, as well as reactive nitrogen species, are produced under physiological conditions during common metabolic pathways. These reactive species act as second messengers and can subsequently influence various signalling pathways positively or negatively, depending on the regulatory mechanism of their concentration, termed redox regulation.^184^ Similarly, mitochondria can produce antioxidants that counteract the deleterious effects of OH to maintain the balance between ROS production and detoxification.^184,185^ On the other hand, pyruvate, an energy metabolic substrate, has been shown to protect against motor neuron loss induced by the excitotoxic effects of AMPA and paralysis, indicating that increased intracellular Ca^2+^ probably interferes with mitochondrial energy metabolism.^98^ Therefore, since pyruvate is a normal metabolite that has no serious side effects, it has been suggested that it may be a neuroprotective agent in sALS.^98^

It has recently been shown that mitochondria and the endoplasmic reticulum (ER) are intertwined through membranes, known as mitochondria-associated membranes (MAM),^173^ and the loss of connection between both organelles is associated with neurodegenerative processes in motor neurons. MAM is a multifunctional microdomain in cellular homeostasis and is recently one of the most relevant structures implicated in the disease. It is one of the current perspectives from which the study of ALS is being focused. In addition, MAM has also been shown to be essential for the regulation of autophagy and mitochondrial dynamics.^186^

The damage induced in neuronal mitochondria and ER by excitotoxicity and increased Ca^2+^ influx favours surrounding astrocytes to promote increased ROS production, as well as promoting the progression of more excitotoxic stress and neurodegeneration.^187-189^ ROS are presumed to play a central role in damage propagation by targeting surrounding glia and increasing motor neuron excitability. This alters mitochondrial activity and causes Ca^2+^ buffering to become ineffective and cytosolic Ca^2+^ levels to increase.^174^ These data are supported by studies showing that oxidative stress and mitochondrial dysfunction in SOD1 mutant astrocytes have the potential to induce motor neuron death and/or contribute to disease propagation.^114^ Furthermore, this oxidative stress and, especially, ONOO formed in reactive astrocytes could cause long-term effects on specific proteins, such as connexins and enzymes, that can drastically affect astrocyte-neuron interactions. In addition, ONOO also inhibits astrocytic glutamate transporters, leading to increased neurotoxic influences on motor neurons through potentiation of neuronal excitability and excitatory neurotransmission.^190,191^

So, the heightened influx of Ca^2+^ leading to excitotoxicity induces strain on both mitochondria and ER, resulting in substantial damage to these intracellular structures. Consequently, this damage contributes to the detriment of the motor neurons, activating cell signalling pathways that lead to motor neuron degeneration and death.

#### Activation of cell death pathways

Normal development and tissue homeostasis in multicellular organisms depend on programmed cell death (PCD) signalling events, which are well-orchestrated and tightly regulated.^192^ Although the elimination of superfluous neuronal cells is vital for normal brain function, defects in neuronal cell PCD signalling, such as apoptosis, necroptosis, pyroptosis, ferroptosis, and the aberrant death of distinct neuronal cell populations associated with autophagy, can be observed in the pathogenesis of various neurological diseases, such as ALS, resulting in unwanted loss of neuronal cells and function.^193-195^

However, are abnormalities in cell death signalling and neuronal loss a direct cause of these diseases or merely a secondary effect of the underlying damage? Additionally, it remains unclear how various PCD pathways and other biological mechanisms work together to contribute to the degeneration of neurons and other cell types in these conditions. These cell death pathways can be activated in response to various forms of cellular stress exerted by intracellular or extracellular stimuli, as well as inflammatory processes. As previously discussed, the aetiology of neurodegenerative diseases is multifactorial, being associated with defects in different cellular processes, such as response to oxidative stress, excitotoxicity, and inflammation.^196,197^ This oxidative stress and neuroinflammation have been shown to be intrinsic components of neurodegeneration associated with deposits of the protein TDP-43.^198^ This protein causes neuronal death due to toxic gain of function, cytoplasmic aggregates, and loss of function, nuclear depletion, and triggers mitochondrial dysfunction in neuronal cells.^199^

It has been shown that this oxidative stress and the neuroinflammation are intrinsic components of TDP-43-associated neurodegeneration, and the balance between the cytoprotective JNK and cytotoxic p38 protein signalling dictates the phenotypic outcome of TDP-43 expression during ALS. P38 mitogen-activated protein kinase (p38MAPK) is one of the key proteins involved in the activation of the cell death pathway and plays a critical role in promoting TDP-43 proteinopathy. Inhibition of the p38α MAPK isoform reduces ALS-associated TDP-43 phenotypes. Thus, p38α inhibition mitigates aberrant TDP-43 phenotypes in motor neurons derived from patients with ALS.^200^

Therefore, the interplay between Ca^2+^ overload, oxidative damage, inflammation processes, and mitochondrial dysfunction contributes to both apoptotic and necrotic cell death in ALS. These processes, along with the involvement of p38 and TDP-43 proteinopathy, lead to motor neuron degeneration. Although abnormal cell death is typical in neurodegenerative diseases, the precise mechanisms remain elusive. Further research using animal models and human samples is essential to develop effective treatments and novel therapeutic approaches.

In summary, the vulnerability of motor neurons to excitotoxicity is due to several intrinsic characteristics, including their high energy demands, large size, limited capacity to buffer intracellular Ca^2+^, reliance on astrocytes for glutamate clearance, and the presence of glutamate receptors that allow Ca^2+^ influx. These factors collectively promote the development of local excitotoxic processes that disrupt lipid metabolism regulation by the ER and mitochondria. This disruption initiates a vicious cycle of neuronal damage and apoptotic signalling, ultimately leading to motor neuron death.

### Strategic therapies targeting excitotoxicity

Considering the diverse factors influencing the onset of ALS and the wide range of symptoms exhibited by patients, identifying a universal therapy capable of halting disease progression remains extremely challenging. To date, therapeutic strategies have focused on modulating glutamate signalling to reduce excitotoxicity, given the strong evidence suggesting that glutamate-induced neuronal damage plays a central role in motor neuron degeneration.^59,182^ Currently, different therapeutic approaches have been tested to slow disease progression and improve patients’ quality of life, including symptom-relieving medications, as well as physical and occupational therapies delivered within a multidisciplinary care framework.^32^ Notably, multidisciplinary management has been shown to significantly prolong patient survival and enhance the effectiveness of specific treatments.^201-204^

Riluzole is a drug classified as a glutamate release inhibitor, aiming to reduce the amount of glutamate released into the synaptic cleft, thereby minimizing overactivation of glutamate receptors.^205^ Among the numerous treatments tested for ALS patients in recent years, riluzole remains the only globally approved therapy and is the most widely used due to its modest yet significant benefits, primarily through inhibiting glutamate release and attenuating excitotoxicity in ALS.^206,207^ Although the benefit demonstrated in the original clinical trial was more modest,^208^ further studies of real-life evidence have shown that patients treated with riluzole exhibited more prolonged median survival than those who did not receive this treatment,^209^ by preserving motor neuron function and attenuating disease progression.^206,207^

Specifically, riluzole acts by inhibiting presynaptic voltage-gated Na^+^ channels, thereby reducing neuronal depolarization and glutamate release. In the context of ALS, depolarization facilitates Ca^2+^ influx into presynaptic terminals, triggering excessive glutamate release. This excess of glutamate overstimulates postsynaptic neurons via glutamatergic receptor activation, contributing to motor neuron injury and degeneration.^210^ By limiting this glutamate overflow, riluzole helps preserve neuronal integrity.

In addition to its effects on presynaptic neurons, riluzole enhances glutamate clearance by upregulating EAAT2 activity in astrocytes,^205,210^ thereby facilitating glutamate reuptake and reducing synaptic glutamate levels. Furthermore, this drug modulates inhibitory neurotransmission by acting on GABAergic pathways. It inhibits the astrocytic reuptake of GABA, prolonging its presence in the synaptic cleft while also enhancing postsynaptic GABA receptor activation.^205^ These combined actions contribute to restoring the excitatory/inhibitory balance disrupted in ALS, reinforcing riluzole’s multifaceted therapeutic profile. So, this treatment helps preserve neuronal integrity and has demonstrated efficacy in preserving motor neuron function and attenuating disease progression.^206,207^

Other treatments, such as ceftriaxone, act as glutamate reuptake enhancers. This agent aims to improve extracellular glutamate clearance by increasing the function or expression of astrocytic glutamate transporters, especially EAAT2 in astrocytes.^211^ Ceftriaxone has been shown to upregulate EAAT2 transcription and protein expression. Preclinical studies by Rothstein *et al*.^212^ demonstrated that ceftriaxone increased EAAT2 levels and delayed symptom onset in ALS mouse models. However, as stated above, a phase III clinical trial (NCT00349622) investigating ceftriaxone in ALS patients was terminated due to lack of efficacy in improving survival, despite promising mechanistic data.^95^ Nevertheless, this study provided proof of concept for pharmacological upregulation of glutamate transporters.

In addition to enhancing glutamate reuptake, another therapeutic avenue involves directly inhibiting ionotropic glutamate receptors (especially AMPA and NMDA receptors) to prevent Ca^2+^ overload and excitotoxicity. These approaches aim to attenuate glutamate-induced neuronal damage at the receptor level.^211^ Drugs such as talampanel and perampanel block AMPA receptors to reduce excitatory synaptic transmission and subsequent Ca^2+^ entry into neurons.^213^ These agents demonstrated neuroprotective effects in preclinical ALS models.^214,215^ However, clinical trials with talampanel were discontinued due to a lack of significant efficacy and tolerability concerns, limiting their clinical application.^216^

On the other hand, NMDA receptor blockers, including memantine and dextromethorphan, reduce Ca^2+^ influx by inhibiting NMDA receptor activation. These drugs have shown limited neuroprotective effects in ALS models.^217^ Memantine, widely used in Alzheimer's disease, has not demonstrated sufficient efficacy in ALS to warrant approval but remains under investigation.^218,219^

Finally, among the drugs currently in the clinical trial for ALS patients, tofersen stands out. Although this therapy does not directly target excitotoxic mechanisms, it indirectly contributes to reducing excitotoxicity by acting on the underlying pathogenic process. This drug has been developed to specifically target *SOD1* mRNA, reducing the synthesis of SOD1 protein, whose mutation causes incorrect folding and grouping, resulting in a gain in toxicity and being key in the development of the disease in patients with fALS with the SOD1 mutation.^220,221^ Administered intrathecally, tofersen has demonstrated efficacy in both preclinical models and clinical trials, significantly lowering SOD1 protein and mRNA levels in the CNS.^222^ Recent studies have also shown that tofersen treatment correlates with a reduction in neurofilament light chain levels in both serum and CSF, an essential biomarker for early neurodegeneration.^222^ In 2023, tofersen was approved in the USA for use in patients with SOD1-linked fALS, being the only treatment to date used, combined with riluzole, in SOD-1 fALS patients.^223^ However, treatment is not without risk, as adverse events such as increased intracranial pressure, lumbar radiculopathy, or myelitis have been reported.^221,224-226^ In 2024, the European Medicines Agency (EMA) granted orphan drug designation to tofersen, because the double-blind, randomized trial did not meet its primary endpoint, despite achieving secondary endpoints, and the drug is currently being evaluated in an open-label (unblinded) extension phase.^227^

#### Future directions

The search for developing effective treatments for ALS continues to be one of the greatest challenges in neurodegenerative disease research. Although numerous compounds have shown promising results in animal models or *in vitro* studies, only a handful have progressed successfully to the clinical stage. A key reason for this persistent gap is the limited translational value of experimental models, such as the *Sod1* mouse model, which represents only a small subset of familial cases and fails to capture the broad clinical and molecular heterogeneity of sALS.^228^ These models often fail to replicate the complex progression and clinical heterogeneity of the disease, limiting their predictive value for human outcomes. Addressing this issue will require the development of more representative systems, such as human neuronal organoids and patient-derived cell lines, that better mimic the genetic and phenotypic diversity observed in patients. Such models could bridge the critical gap between laboratory research and clinical application, improving both predictive accuracy and therapeutic relevance.

At the same time, current research in ALS is undergoing a conceptual shift. The goal is no longer to slow disease progression, but to halt, or ideally, reverse the neuronal loss. This shift demands a multifaceted approach capable of tackling the complex and overlapping mechanisms that drive ALS, including excitotoxicity, oxidative stress, and inflammation. It also highlights the importance of more sensitive diagnostic tools that allow earlier detection and intervention, when motor neurons may still be rescued. The integration of molecular biology, clinical neurology, and precision medicine will be central to this effort, enabling individualized treatments based on each patient’s unique genetic and molecular profile.

Among emerging strategies, those focused on modulating glutamate homeostasis have gained considerable attention. Combination therapies that simultaneously target glutamate excitotoxicity, neuroinflammation, and oxidative stress may offer synergistic benefits compared with single-agent approaches.

In parallel, precision medicine initiatives seeking to identify the subpopulations most likely to benefit from EAAT2 modulation or receptor antagonism could increase the likelihood of clinical success, and provide an important demonstration of the importance of restoring excess glutamate. This possibility of enhancing astrocytic glutamate uptake has already been explored.^64^ Advances in delivery systems, such as nanoparticles or astrocyte-targeted vectors, might also help overcome the pharmacokinetic limitations that have hindered previous candidates.^229^ A particularly innovative line of research involves the regulation of glutamate signalling at the gene expression level. Antisense oligonucleotides directed against EAAT2, designed to enhance its expression or restore its transport activity in astrocytes, have shown encouraging results in preclinical models. Related work using gene therapy approaches has demonstrated that modulating astrocytic uptake capacity can mitigate excitotoxic damage. Additionally, experiments in animal models have demonstrated that microRNA-218, which is highly enriched in motor neurons, can be taken up by astrocytes, inducing in them a downregulation of the EAAT2 glutamate transporter, and it has seen that the inhibition of miR-218 mitigates EAAT2 loss and astrogliosis.^230^ Together, these findings suggest that RNA-based interventions, although remains in preclinical development, could provide a more precise and durable means of restoring glutamate balance.^64,230^

Nevertheless, several obstacles remain before these strategies can be translated into clinical benefit. Limited permeability of the blood–brain barrier, possible off-target effects associated with broad glutamate modulation, and variability among patients continue to complicate therapeutic development. Moreover, as excitotoxicity is intimately linked with oxidative stress, mitochondrial dysfunction, and neuroinflammation, restoring glutamate balance alone is unlikely to suffice.

In conclusion, advancing ALS treatment, targeting the excitotoxic processes, will depend on the convergence of more predictive experimental models, precision-based therapeutic design, and multimodal intervention strategies. Enhancing glutamate reuptake through pharmacological or genetic upregulation of EAAT2 remains one of the most promising avenues under investigation as a therapeutic strategy for ALS. Its eventual success will require not only technological refinement, but also a deep integration between basic and clinical research, an approach essential for translating experimental progress into tangible benefits for patients, improving the prognosis and quality of life for individuals affected by this devastating disorder. However, this will require a multifaceted approach that addresses the complex and overlapping pathogenic mechanisms of ALS. Continued refinement of delivery methods and combination with complementary neuroprotective approaches will be essential to realize its clinical potential. Equally important is the integration of basic and clinical research, along with the implementation of personalized medicine strategies that consider the unique genetic, molecular, and clinical profiles of each patient.

## Conclusion

Excitotoxicity represents a central pathogenic process in ALS, linking molecular and metabolic disturbances that lead to motor neuron death. Compelling evidence indicates that an excessive glutamate release, paralleled by impaired glutamate clearance, primarily due to the dysfunction of astrocytic EATT2 transporters, leads to excessive extracellular glutamate accumulation and aberrant glutamatergic neurotransmission. This results in the overactivation of ionotropic and metabotropic receptors, excessive Ca^2+^ influx, and subsequent oxidative and mitochondrial dysfunction, neurofilament abnormalities, protein aggregation, axonal transport impairments, and ultimately, activation of pathways triggering apoptosis.^53^ These alterations are further exacerbated by glial activation and neuroinflammation, which perpetuate a self-reinforcing cycle of excitotoxic injury. This complexity underscores why ALS remains challenging to diagnose and treat, lacking definitive diagnostic tests or procedures.

As previously explained, ALS is characterized by the fact that specific populations of motor neurons are more resistant to neurodegeneration, while others are more vulnerable and prone to degeneration. The distinct vulnerability is partly linked to the ability to buffer excess Ca^2+^ within neurons in response to oxidative stress. Excessive glutamate transmission triggers a harmful influx of Ca^2+^, which leads to destructive processes, neurodegeneration and ultimately, cell death. Therefore, the differential expression of glutamate receptors, highly permeable to Ca^2+^, along with the presence of Ca^2+^-buffering proteins, determines the natural capacity of neurons to manage Ca^2+^ levels, explaining, at least in part, why some motor neurons are more resistant than others.

Despite various contributing factors, one of the most prominent mechanisms involved in ALS appears to be the excessive production of harmful ROS, which disrupt glutamate transport in the CNS and lead to increased glutamate levels. The result is a vicious, self-perpetuating cycle of cellular damage, affecting both motor neurons and glial cells, contributing to the observed impairment of glutamate homeostasis and increased excitotoxicity, which are key features observed in ALS.

Emerging evidence highlights the multifaceted role of glial cells, particularly astrocytes, in the pathogenesis and progression of ALS. Elevated levels of glutamate in the brain and reduction of EAAT2 transporters in astrocytes, resulting from the *SOD1* mutation, contribute to motor neuron degeneration, damage to neighbouring cells, and eventual paralysis. Additionally, glial cells overexpressing the SOD1 mutation negatively affect the viability of spinal motor neurons by increasing extracellular glutamate levels, which accelerates motoneuronal degeneration.^231^ This explains why many patients with ALS have high levels of glutamate in the CSF and a selective reduction of the astrocytic glutamate transporter EAAT2, coinciding with the appearance of astrogliosis and supporting the excitotoxic hypothesis of motor neuron degeneration. The involvement of EAAT2 receptors in the onset and progression of the disease is so critical that nearly all treatments developed to date have targeted these receptors to prevent their saturation by excess glutamate. Notably, the only drug to show beneficial effects thus far, riluzole, acts on EAAT2 receptors.

Concurrently, microglia further exacerbate this toxic environment through non-vesicular glutamate release and the secretion of proinflammatory mediators, which in turn induce the formation of neurotoxic reactive astrocytes. The bidirectional crosstalk between astrocytes and microglia amplifies neuroinflammation, other motor neuron degeneration.

In addition, mitochondrial and ER dysfunctions have emerged as pivotal contributors to ALS pathogenesis, particularly through their roles in Ca^2+^ homeostasis, lipid metabolism, and oxidative stress regulation. Motor neurons exhibit heightened vulnerability to excitotoxic Ca^2+^ influx, which overwhelms mitochondrial buffering capacity, leading to ROS overproduction and bioenergetics failure. These organelle stresses also intersect with dysregulated PCD pathways further contributing to neuronal loss. These alterations, along with glial contributions, exacerbate excitotoxicity and motor neuron degeneration, positioning organelle stress and redox imbalance as key therapeutic targets.

A key objective in ALS research is to identify specific and quantifiable biomarkers that can confirm early diagnosis, enabling the timely initiation of palliative treatments. A significant obstacle in the development of effective treatments for ALS is the persistent challenge of translating promising preclinical outcomes into tangible clinical benefits. Although numerous therapeutic candidates demonstrate efficacy in animal models or *in vitro* systems, the majority fail in later-stage clinical trials. The development of new and more representative study models is essential for validating potential treatments in clinical settings, leading to more promising outcomes and ensuring broader applicability across all ALS subtypes. Addressing this challenge is urgent, and given the lack of curative therapies, the creation of novel treatments capable of modulating this rapidly progressive and fatal disease is of paramount importance.

This review has aimed to explore the key excitotoxic mechanisms and events involved in the ALS, emphasizing the roles of glutamate receptors, EATT2 transporters, and Ca^2+^-buffering proteins in promoting neuronal survival by clearing excess glutamate and regulating intracellular Ca^2+^ levels, two processes whose dysregulated, contributes significantly to the initiation and progression of the neurodegeneration. A gaining a deeper understanding of the specific molecular causes that lead to excitotoxicity and motor neuron degeneration is essential. Targeting excess glutamate and Ca^2+^ overload represents a promising strategy to slow disease progression. Emerging therapies, such as enhancing astrocyte function or modulating glutamate receptor activity, aim to restore glutamate homeostasis, protect motor neurons, and mitigate the oxidative stress, potentially slowing the progression of neurodegeneration and improving patient life expectancy.

## Data Availability

Data sharing is not applicable to this article as no new data were created or analysed in this study.

## References

[1] Boillée S, Vande Velde C, Cleveland DW. ALS: A disease of motor neurons and their nonneuronal neighbors. Neuron. 2006;52(1):39–59. 17015226 10.1016/j.neuron.2006.09.018

[2] Boillée S, Yamanaka K, Lobsiger CS, et al Onset and progression in inherited ALS determined by motor neurons and microglia. Science. 2006;312(5778):1389–1392. 16741123 10.1126/science.1123511

[3] Lobsiger CS, Boillée S, Cleveland DW. Toxicity from different SOD1 mutants dysregulates the complement system and the neuronal regenerative response in ALS motor neurons. Proc Natl Acad Sci U S A. 2007;104(18):7319–7326. 17463094 10.1073/pnas.0702230104 PMC1863491

[4] Luna J, Diagana M, Ait Aissa L, et al Clinical features and prognosis of amyotrophic lateral sclerosis in Africa: The TROPALS study. J Neurol Neurosurg Psychiatry. 2019;90(1):20–29. 30242088 10.1136/jnnp-2018-318469

[5] Green SL, Tolwani RJ. Animal models for motor neuron disease. Neurology. 1999;49(5):480–487. 10551448

[6] Logroscino G, Traynor BJ, Hardiman O, et al Incidence of amyotrophic lateral sclerosis in Europe. J Neurol Neurosurg Psychiatry. 2010;81(4):385–390. 19710046 10.1136/jnnp.2009.183525 PMC2850819

[7] Marin B, Fontana A, Arcuti S, et al Age-specific ALS incidence: A dose–response meta-analysis. Eur J Epidemiol. 2018;33(7):621–634. 29687175 10.1007/s10654-018-0392-x

[8] Mehta P, Kaye W, Raymond J, et al Prevalence of amyotrophic lateral sclerosis—United States, 2015. MMWR Morb Mortal Wkly Rep. 2018;67(46):1285–1289. 30462626 10.15585/mmwr.mm6746a1 PMC6289079

[9] Goutman SA, Hardiman O, Al-Chalabi A, et al Recent advances in the diagnosis and prognosis of amyotrophic lateral sclerosis. Lancet Neurol. 2022;21(5):480–493. 35334233 10.1016/S1474-4422(21)00465-8 PMC9513753

[10] Chen S, Sayana P, Zhang X, Le W. Genetics of amyotrophic lateral sclerosis: An update. Mol Neurodegener. 2013;8(1):28. 23941283 10.1186/1750-1326-8-28 PMC3766231

[11] Di Giorgio FP, Boulting GL, Bobrowicz S, Eggan KC. Human embryonic stem cell-derived motor neurons are sensitive to the toxic effect of glial cells carrying an ALS-causing mutation. Cell Stem Cell. 2008;3(6):637–648. 19041780 10.1016/j.stem.2008.09.017

[12] Taylor JP, Brown RH, Cleveland DW. Decoding ALS: From genes to mechanism. Nature. 2016;539(7628):197–206. 27830784 10.1038/nature20413 PMC5585017

[13] Hardiman O, Al-Chalabi A, Chio A, et al Amyotrophic lateral sclerosis. Nat Rev Dis Primers. 2017;3:17071. 28980624 10.1038/nrdp.2017.71

[14] Riancho J, Gonzalo I, Ruiz-Soto M, Berciano J. Why do motor neurons degenerate? Actualization in the pathogenesis of amyotrophic lateral sclerosis. Neurologia. 2019;34(1):27–37. 26853842 10.1016/j.nrl.2015.12.001

[15] Chancello AM, Warlow CP. Adult onset motor neuron disease: Worldwide mortality, incidence and distribution since 1950. J Neurol Neurosurg Psychiatry. 1992;55(12):1106–1115. 1479386 10.1136/jnnp.55.12.1106 PMC1015320

[16] Walhout R, Verstraete E, van den Heuvel MP, Veldink JH, van den Berg LH. Patterns of symptom development in patients with motor neuron disease. Amyotroph Lateral Scler Front Degener. 2018;19(1-2):21–28. 10.1080/21678421.2017.1386688 29037065

[17] Kwong LK, Neumann M, Sampathu DM, Lee VMY, Trojanowski JQ. TDP-43 proteinopathy: The neuropathology underlying major forms of sporadic and familial frontotemporal lobar degeneration and motor neuron disease. Acta Neuropathol. 2007;114(1):63–70. 17492294 10.1007/s00401-007-0226-5

[18] Feldman EL, Goutman SA, Petri S, et al Amyotrophic lateral sclerosis. Lancet. 2022;400(10360):1363–1380. 36116464 10.1016/S0140-6736(22)01272-7 PMC10089700

[19] Chiò A, Calvo A, Moglia C, et al Phenotypic heterogeneity of amyotrophic lateral sclerosis: A population based study. J Neurol Neurosurg Psychiatry. 2011;82(7):740–746. 21402743 10.1136/jnnp.2010.235952

[20] Al-Chalabi A, Hardiman O, Kiernan MC, Chiò A, Rix-Brooks B, van den Berg LH. Amyotrophic lateral sclerosis: Moving towards a new classification system. Lancet Neurol. 2016;15(11):1182–1194. 27647646 10.1016/S1474-4422(16)30199-5

[21] Ryan M, Heverin M, McLaughlin RL, Hardiman O. Lifetime risk and heritability of amyotrophic lateral sclerosis. JAMA Neurol. 2019;76(11):1367–1374. 31329211 10.1001/jamaneurol.2019.2044 PMC6646974

[22] Abramzon YA, Fratta P, Traynor BJ, Chia R. The overlapping genetics of amyotrophic lateral sclerosis and frontotemporal dementia. Front Neurosci. 2020;14:42. 32116499 10.3389/fnins.2020.00042 PMC7012787

[23] Balendra R, Isaacs AM. C9orf72-mediated ALS and FTD: Multiple pathways to disease. Nat Rev Neurol. 2018;14(9):544–558. 30120348 10.1038/s41582-018-0047-2 PMC6417666

[24] Ferrari R, Kapogiannis D, Huey ED, Momeni P. FTD and ALS: A tale of two diseases. Curr Alzheimer Res. 2011;8(3):273–294. 21222600 10.2174/156720511795563700 PMC3801195

[25] DeJesus-Hernandez M, Mackenzie IR, Boeve BF, et al Expanded GGGGCC hexanucleotide repeat in noncoding region of C9ORF72 causes chromosome 9p-linked FTD and ALS. Neuron. 2011;72(2):245–256. 21944778 10.1016/j.neuron.2011.09.011 PMC3202986

[26] Renton AE, Majounie E, Waite A, et al A hexanucleotide repeat expansion in C9ORF72 is the cause of chromosome 9p21-linked ALS-FTD. Neuron. 2011;72(2):257–268. 21944779 10.1016/j.neuron.2011.09.010 PMC3200438

[27] Murphy NA, Arthur KC, Tienari PJ, Houlden H, Chiò A, Traynor BJ. Age-related penetrance of the C9orf72 repeat expansion. Sci Rep. 2017;7(1):2116. 28522837 10.1038/s41598-017-02364-1 PMC5437033

[28] Kaur SJ, McKeown SR, Rashid S. Mutant SOD1 mediated pathogenesis of amyotrophic lateral sclerosis. Gene. 2016;577(2):109–118. 26657039 10.1016/j.gene.2015.11.049

[29] Andersen PM, Sims KB, Xin WW, et al Sixteen novel mutations in the Cu/Zn superoxide dismutase gene in amyotrophic lateral sclerosis: A decade of discoveries, defects and disputes. Amyotroph Lateral Scler Other Mot Neuron Disord. 2003;4(2):62–73. 10.1080/14660820310011700 14506936

[30] Rosen DR, Siddique T, Patterson D, et al Mutations in Cu/Zn superoxide dismutase gene are associated with familial amyotrophic lateral sclerosis. Nature. 1993;362(6415):59–62. 8446170 10.1038/362059a0

[31] Tsou YS, Lai JH, Chen KY, Chang CF, Huang CC. Therapeutic effect of rapamycin on TDP-43-related pathogenesis in ischemic stroke. Int J Mol Sci. 2023;24(1):676. 10.3390/ijms24010676 PMC9820757 36614118

[32] Corcia P, Lunetta C, Vourc’h P, Pradat PF, Blasco H. Time for optimism in amyotrophic lateral sclerosis. Eur J Neurol. 2023;30(5):1459–1464. 36773012 10.1111/ene.15738

[33] Peters OM, Ghasemi M, Brown RH. Emerging mechanisms of molecular pathology in ALS. J Clin Invest. 2015;125(5):1767–1779. 25932674 10.1172/JCI71601 PMC4463186

[34] Greaves CV, Rohrer JD. An update on genetic frontotemporal dementia. J Neurol. 2019;266(8):2075–2086. 31119452 10.1007/s00415-019-09363-4 PMC6647117

[35] Mahoney CJ, Beck J, Rohrer JD, et al Frontotemporal dementia with the C9ORF72 hexanucleotide repeat expansion: Clinical, neuroanatomical and neuropathological features. Brain. 2012;135(3):736–750. 22366791 10.1093/brain/awr361 PMC3286330

[36] Zarei S, Carr K, Reiley L, et al A comprehensive review of amyotrophic lateral sclerosis. Surg Neurol Int. 2015;6(1):171. 26629397 10.4103/2152-7806.169561 PMC4653353

[37] Kiriyama Y, Nochi H. The function of autophagy in neurodegenerative diseases. Int J Mol Sci. 2015;16(11):26797–26812. 26569220 10.3390/ijms161125990 PMC4661849

[38] Maruyama H, Morino H, Ito H, et al Mutations of optineurin in amyotrophic lateral sclerosis. Nature. 2010;465(7295):223–226. 20428114 10.1038/nature08971

[39] Kao AW, McKay A, Singh PP, Brunet A, Huang EJ. Progranulin, lysosomal regulation and neurodegenerative disease. Nat Rev Neurosci. 2017;18(6):325–333. 28435163 10.1038/nrn.2017.36 PMC6040832

[40] Karamysheva ZN, Tikhonova EB, Karamyshev AL. Granulin in frontotemporal lobar degeneration: Molecular mechanisms of the disease. Front Neurosci. 2019;13(APR):395. 31105517 10.3389/fnins.2019.00395 PMC6494926

[41] Vance C, Rogelj B, Hortobágyi T, et al Mutations in FUS, an RNA processing protein, cause familial amyotrophic lateral sclerosis type 6. Science. 2009;323(5918):1208–1211. 19251628 10.1126/science.1165942 PMC4516382

[42] Kwiatkowski TJ, Bosco DA, LeClerc AL, et al Mutations in the *FUS/TLS* gene on chromosome 16 cause familial amyotrophic lateral sclerosis. Science. 2009;323(5918):1205–1208. 19251627 10.1126/science.1166066

[43] Dadon-Nachum M, Melamed E, Offen D. The “dying-back” phenomenon of motor neurons in ALS. J Mol Neurosci. 2011;43(3):470–477. 21057983 10.1007/s12031-010-9467-1

[44] Dupuis L, Gonzalez de Aguilar JL, Echaniz-Laguna A, et al Muscle mitochondrial uncoupling dismantles neuromuscular junction and triggers distal degeneration of motor neurons. PLoS One. 2009;4(4):e5390. 19404401 10.1371/journal.pone.0005390 PMC2671839

[45] Fischer LR, Culver DG, Tennant P, et al Amyotrophic lateral sclerosis is a distal axonopathy: Evidence in mice and man. Exp Neurol. 2004;185(2):232–240. 14736504 10.1016/j.expneurol.2003.10.004

[46] Frey D, Schneider C, Xu L, Borg J, Spooren W, Caroni P. Early and selective loss of neuromuscular synapse subtypes with low sprouting competence in motoneuron diseases. J Neurosci. 2000;20(7):2534–2542. 10729333 10.1523/JNEUROSCI.20-07-02534.2000 PMC6772256

[47] Rocha MC, Pousinha PA, Correia AM, Sebastião AM, Ribeiro JA. Early changes of neuromuscular transmission in the SOD1(G93A) mice model of ALS start long before motor symptoms onset. PLoS One. 2013;8(9):e73846. 24040091 10.1371/journal.pone.0073846 PMC3764017

[48] Haenggeli C, Kato AC. Differential vulnerability of cranial motoneurons in mouse models with motor neuron degeneration. Neurosci Lett. 2002;335(1):39–43. 12457737 10.1016/s0304-3940(02)01140-0

[49] Nijssen J, Comley LH, Hedlund E. Motor neuron vulnerability and resistance in amyotrophic lateral sclerosis. Acta Neuropathol. 2017;133(6):863–885. 28409282 10.1007/s00401-017-1708-8 PMC5427160

[50] Nimchinsky EA, Young WG, Yeung G, et al Differential vulnerability of oculomotor, facial, and hypoglossal nuclei in G86R superoxide dismutase transgenic mice. J Comp Neurol. 2000;416(1):112–125. 10578106 10.1002/(sici)1096-9861(20000103)416:1<112::aid-cne9>3.0.co;2-k

[51] Silva-Hucha S, de Sevilla ME F, Humphreys KM, et al VEGF expression disparities in brainstem motor neurons of the SOD1G93A ALS model: Correlations with neuronal vulnerability. Neurotherapeutics. 2024;21(3):e00340. 38472048 10.1016/j.neurot.2024.e00340 PMC11070718

[52] Lafarga V, Sirozh O, Díaz-López I, et al Widespread displacement of DNA- and RNA-binding factors underlies toxicity of arginine-rich cell-penetrating peptides. EMBO J. 2021;40(13):e103311. 33978236 10.15252/embj.2019103311 PMC8246256

[53] Gould TW, Oppenheim RW. Motor neuron trophic factors: Therapeutic use in ALS? Brain Res Rev. 2011;67(1-2):1–39. 20971133 10.1016/j.brainresrev.2010.10.003 PMC3109102

[54] Avossa D, Grandolfo M, Mazzarol F, Zatta M, Ballerini L. Early signs of motoneuron vulnerability in a disease model system: Characterization of transverse slice cultures of spinal cord isolated from embryonic ALS mice. Neuroscience. 2006;138(4):1179–1194. 16442737 10.1016/j.neuroscience.2005.12.009

[55] Carunchio I, Mollinari C, Pieri M, Merlo D, Zona C. GABAA receptors present higher affinity and modified subunit composition in spinal motor neurons from a genetic model of amyotrophic lateral sclerosis. Eur J Neurosci. 2008;28(7):1275–1285. 18973555 10.1111/j.1460-9568.2008.06436.x

[56] Cleveland DW, Rothstein JD. From Charcot to Lou Gehrig: Deciphering selective motor neuron death in ALS. Nat Rev Neurosci. 2001;2(11):806–819. 11715057 10.1038/35097565

[57] Pasinelli P, Brown RH. Molecular biology of amyotrophic lateral sclerosis: Insights from genetics. Nat Rev Neurosci. 2006;7(9):710–723. 16924260 10.1038/nrn1971

[58] Wu WL, Gong XX, Qin ZH, Wang Y. Molecular mechanisms of excitotoxicity and their relevance to the pathogenesis of neurodegenerative diseases—An update. Acta Pharmacol Sin. 2025;46(12):3129–3142. 40389567 10.1038/s41401-025-01576-w PMC12644896

[59] de Araújo Boleti AP, de Oliveira Flores TM, Moreno SE, dos Anjos L, Mortari MR, Migliolo L. Neuroinflammation: An overview of neurodegenerative and metabolic diseases and of biotechnological studies. Neurochem Int. 2020;136:104714. 32165170 10.1016/j.neuint.2020.104714

[60] Heath PR, Shaw PJ. Update on the glutamatergic neurotransmitter system and the role of excitotoxicity in amyotrophic lateral sclerosis. Muscle Nerve. 2002;26(4):438–458. 12362409 10.1002/mus.10186

[61] Raiteri L, Stigliani S, Zappettini S, Mercuri NB, Raiteri M, Bonanno G. Excessive and precocious glutamate release in a mouse model of amyotrophic lateral sclerosis. Neuropharmacology. 2004;46(6):782–792. 15033338 10.1016/j.neuropharm.2003.11.025

[62] Milanese M, Zappettini S, Onofri F, et al Abnormal exocytotic release of glutamate in a mouse model of amyotrophic lateral sclerosis. J Neurochem. 2011;116(6):1028–1042. 21175617 10.1111/j.1471-4159.2010.07155.x

[63] Bonifacino T, Musazzi L, Milanese M, et al Altered mechanisms underlying the abnormal glutamate release in amyotrophic lateral sclerosis at a pre-symptomatic stage of the disease. Neurobiol Dis. 2016;95:122–133. 27425885 10.1016/j.nbd.2016.07.011

[64] Grad LI, Rouleau GA, Ravits J, Cashman NR. Clinical spectrum of amyotrophic lateral sclerosis (ALS). Cold Spring Harb Perspect Med. 2017;7(8):a024117. 28003278 10.1101/cshperspect.a024117 PMC5538408

[65] King AE, Woodhouse A, Kirkcaldie MTK, Vickers JC. Excitotoxicity in ALS: Overstimulation, or overreaction? Exp Neurol. 2016;275(2016):162–171. 26584004 10.1016/j.expneurol.2015.09.019

[66] Rothstein JD, Van Kammen M, Levey AI, Martin LJ, Kuncl RW. Selective loss of glial glutamate transporter GLT-1 in amyotrophic lateral sclerosis. Ann Neurol. 1995;38(1):73–84. 7611729 10.1002/ana.410380114

[67] Van Damme P, Robberecht W, Van Den Bosch L. Modelling amyotrophic lateral sclerosis: Progress and possibilities. Dis Model Mech. 2017;10(5):537–549. 28468939 10.1242/dmm.029058 PMC5451175

[68] Philips T, Rothstein JD. Glial cells in amyotrophic lateral sclerosis. Exp Neurol. 2014;262:111–120. 24859452 10.1016/j.expneurol.2014.05.015 PMC4241182

[69] Milanese M, Bonifacino T, Zappettini S, et al Glutamate release from astrocytic gliosomes under physiological and pathological conditions. Int Rev Neurobiol. 2009;85: 295–318. 19607977 10.1016/S0074-7742(09)85021-6

[70] Mohamed LA, Markandaiah SS, Bonanno S, Pasinelli P, Trotti D. Excess glutamate secreted from astrocytes drives upregulation of P-glycoprotein in endothelial cells in amyotrophic lateral sclerosis. Exp Neurol. 2019;316:27–38. 30974102 10.1016/j.expneurol.2019.04.002 PMC6506236

[71] Odierna GL, Vucic S, Dyer M, Dickson T, Woodhouse A, Blizzard C. How do we get from hyperexcitability to excitotoxicity in amyotrophic lateral sclerosis? Brain. 2024;147(5):1610–1621. 38408864 10.1093/brain/awae039 PMC11068114

[72] Bonifacino T, Rebosio C, Provenzano F, et al Enhanced function and overexpression of metabotropic glutamate receptors 1 and 5 in the spinal cord of the SOD1G93A mouse model of amyotrophic lateral sclerosis during disease progression. Int J Mol Sci. 2019;20(18):4552. 31540330 10.3390/ijms20184552 PMC6774337

[73] Tortarolo M, Grignaschi G, Calvaresi N, et al Glutamate AMPA receptors change in motor neurons of SOD1G93A transgenic mice and their inhibition by a noncompetitive antagonist ameliorates the progression of amytrophic lateral sclerosis-like disease. J Neurosci Res. 2006;83(1):134–146. 16323214 10.1002/jnr.20715

[74] Kawahara Y, Ito K, Sun H, Aizawa H, Kanazawa I, Kwak S. RNA editing and death of motor neurons. Nature. 2004;427(6977):801. 14985749 10.1038/427801a

[75] Berger JV, Dumont AO, Focant MC, et al Opposite regulation of metabotropic glutamate receptor 3 and metabotropic glutamate receptor 5 by inflammatory stimuli in cultured microglia and astrocytes. Neuroscience. 2012;205:29–38. 22245498 10.1016/j.neuroscience.2011.12.044

[76] Bosch L VD, Van Damme P, Bogaert E, Robberecht W. The role of excitotoxicity in the pathogenesis of amyotrophic lateral sclerosis. Biochim Biophys Acta Mol Basis Dis. 2006;1762(11-12):1068–1082. 10.1016/j.bbadis.2006.05.002 16806844

[77] Carriedo SG, Sensi SL, Yin HZ, Weiss JH. AMPA exposures induce mitochondrial Ca(2+) overload and ROS generation in spinal motor neurons *in vitro*. J Neurosci. 2000;20(1):240–250. 10627601 10.1523/JNEUROSCI.20-01-00240.2000 PMC6774118

[78] Lai TW, Zhang S, Wang YT. Excitotoxicity and stroke: Identifying novel targets for neuroprotection. Prog Neurobiol. 2014;115(C):157–188. 24361499 10.1016/j.pneurobio.2013.11.006

[79] Rogujski P, Lukomska B, Janowski M, Stanaszek L. Glial-restricted progenitor cells: A cure for diseased brain? Biol Res. 2024;57(1):8. 38475854 10.1186/s40659-024-00486-1 PMC10935984

[80] Stoklund Dittlau K, Freude K. Astrocytes: The stars in neurodegeneration? Biomolecules. 2024;14(3):289. 38540709 10.3390/biom14030289 PMC10967965

[81] Galea E, Weinstock LD, Larramona-Arcas R, et al Multi-transcriptomic analysis points to early organelle dysfunction in human astrocytes in Alzheimer’s disease. Neurobiol Dis. 2022;166:105655. 35143967 10.1016/j.nbd.2022.105655 PMC9504227

[82] Jiwaji Z, Tiwari SS, Avilés-Reyes RX, et al Reactive astrocytes acquire neuroprotective as well as deleterious signatures in response to Tau and Aß pathology. Nat Commun. 2022;13(1):135. 35013236 10.1038/s41467-021-27702-w PMC8748982

[83] Verkhratsky A, Butt A, Li B, et al Astrocytes in human central nervous system diseases: A frontier for new therapies. Signal Transduct Target Ther. 2023;8(1):396. 37828019 10.1038/s41392-023-01628-9 PMC10570367

[84] Ridet JL, Malhotra SK, Privat A, Gage FH. Reactive astrocytes: Cellular and molecular cues to biological function. Trends Neurosci. 1997;20(12):570–577. 9416670 10.1016/s0166-2236(97)01139-9

[85] Wang L, Gutmann DH, Roos RP. Astrocyte loss of mutant SOD1 delays ALS disease onset and progression in G85R transgenic mice. Hum Mol Genet. 2011;20(2):286–293. 20962037 10.1093/hmg/ddq463

[86] Lasiene J, Yamanaka K. Glial cells in amyotrophic lateral sclerosis. Neurol Res Int. 2011;2011:718987. 21766027 10.1155/2011/718987 PMC3135155

[87] Yamanaka K, Chun SJ, Boillee S, et al Astrocytes as determinants of disease progression in inherited amyotrophic lateral sclerosis. Nat Neurosci. 2008;11(3):251–253. 18246065 10.1038/nn2047 PMC3137510

[88] Bowman CL, Kimelberg HK. Excitatory amino acids directly depolarize rat brain astrocytes in primary culture. Nature. 1984;311(5987):656–659. 6148706 10.1038/311656a0

[89] Kettenmann H, Schachner M. Pharmacological properties of γ-aminobutyric acid-, glutamate-, and asparatate-induced depolarizations in cultured astrocytes. J Neurosci. 1985;5(12):3295–3301. 2867131 10.1523/JNEUROSCI.05-12-03295.1985 PMC6565228

[90] Provenzano F, Torazza C, Bonifacino T, Bonanno G, Milanese M. The key role of astrocytes in amyotrophic lateral sclerosis and their commitment to glutamate excitotoxicity. Int J Mol Sci. 2023;24(20):15430. 37895110 10.3390/ijms242015430 PMC10607805

[91] Sasaki S, Komori T, Iwata M. Excitatory amino acid transporter 1 and 2 immunoreactivity in the spinal cord in amyotrophic lateral sclerosis. Acta Neuropathol. 2000;100(2):138–144. 10963360 10.1007/s004019900159

[92] Spreux-Varoquaux O, Bensimon G, Lacomblez L, et al Glutamate levels in cerebrospinal fluid in amyotrophic lateral sclerosis: A reappraisal using a new HPLC method with coulometric detection in a large cohort of patients. J Neurol Sci. 2002;193(2):73–78. 11790386 10.1016/s0022-510x(01)00661-x

[93] Deitch JS, Alexander GM, Del Valle L, Heiman-Patterson TD. GLT-1 glutamate transporter levels are unchanged in mice expressing G93A human mutant SOD1. J Neurol Sci. 2002;193(2):117–126. 11790392 10.1016/s0022-510x(01)00656-6

[94] Berry JD, Shefner JM, Conwit R, et al Design and initial results of a multi-phase randomized trial of ceftriaxone in amyotrophic lateral sclerosis. PLoS One. 2013;8(4):e61177. 23613806 10.1371/journal.pone.0061177 PMC3629222

[95] Cudkowicz ME, Titus S, Kearney M, et al Safety and efficacy of ceftriaxone for amyotrophic lateral sclerosis: A multi-stage, randomised, double-blind, placebo-controlled trial. Lancet Neurol. 2014;13(11):1083–1091. 25297012 10.1016/S1474-4422(14)70222-4 PMC4216315

[96] Vergouts M, Doyen PJ, Peeters M, Opsomer R, Hermans E. Constitutive downregulation protein kinase C epsilon in hSOD1G93A astrocytes influences mGluR5 signaling and the regulation of glutamate uptake. Glia. 2018;66(4):749–761. 29266405 10.1002/glia.23279

[97] Bano D, Young KW, Guerin CJ, et al Cleavage of the plasma membrane Na+/Ca^2+^ exchanger in excitotoxicity. Cell. 2005;120(2):275–285. 15680332 10.1016/j.cell.2004.11.049

[98] Corona JC, Tapia R. Ca2+-permeable AMPA receptors and intracellular Ca^2+^ determine motoneuron vulnerability in rat spinal cord in vivo. Neuropharmacology. 2007;52(5):1219–1228. 17320918 10.1016/j.neuropharm.2006.12.008

[99] Nicholls DG, Budd SL. Mitochondria and neuronal survival. Physiol Rev. 2000;80(1):315–360. 10617771 10.1152/physrev.2000.80.1.315

[100] OuYang YB, Kristián T, Kristiánová V, Mellergård P, Siesjö BK. The influence of calcium transients on intracellular pH in cortical neurons in primary culture. Brain Res. 1995;676(2):307–313. 7614000 10.1016/0006-8993(95)00056-v

[101] Van Damme P, Bogaert E, Dewil M, et al Astrocytes regulate GluR2 expression in motor neurons and their vulnerability to excitotoxicity. Proc Natl Acad Sci U S A. 2007;104(37):14825–14830. 17804792 10.1073/pnas.0705046104 PMC1976195

[102] Vargas MR, Johnson JA. Astrogliosis in amyotrophic lateral sclerosis: Role and therapeutic potential of astrocytes. Neurotherapeutics. 2010;7(4):471–481. 20880509 10.1016/j.nurt.2010.05.012 PMC2967019

[103] Bataveljić D, Nikolić L, Milosević M, Todorović N, Andjus PR. Changes in the astrocytic aquaporin-4 and inwardly rectifying potassium channel expression in the brain of the amyotrophic lateral sclerosis SOD1G93A rat model. Glia. 2012;60(12):1991–2003. 22987392 10.1002/glia.22414

[104] Kaiser M, Maletzki I, Hülsmann S, et al Progressive loss of a glial potassium channel (KCNJ10) in the spinal cord of the SOD1 (G93A) transgenic mouse model of amyotrophic lateral sclerosis. J Neurochem. 2006;99(3):900–912. 16925593 10.1111/j.1471-4159.2006.04131.x

[105] Andersen J V, Schousboe A. Milestone review: Metabolic dynamics of glutamate and GABA mediated neurotransmission—The essential roles of astrocytes. J Neurochem. 2023;166(2):109–137. 36919769 10.1111/jnc.15811

[106] Vesce S, Rossi D, Brambilla L, Volterra A. Glutamate release from astrocytes in physiological conditions and in neurodegenerative disorders characterized by neuroinflammation. Int Rev Neurobiol. 2007;82:57–71. 17678955 10.1016/S0074-7742(07)82003-4

[107] Beers DR, Henkel JS, Xiao Q, et al Wild-type microglia extend survival in PU.1 knockout mice with familial amyotrophic lateral sclerosis. Proc Natl Acad Sci U S A. 2006;103(43):16021–16026. 17043238 10.1073/pnas.0607423103 PMC1613228

[108] West M, Mhatre M, Ceballos A, et al The arachidonic acid 5-lipoxygenase inhibitor nordihydroguaiaretic acid inhibits tumor necrosis factor α activation of microglia and extends survival of G93A-SOD1 transgenic mice. J Neurochem. 2004;91(1):133–143. 15379894 10.1111/j.1471-4159.2004.02700.x

[109] Appel SH, Simpson EP. Activated microglia: The silent executioner in neurodegenerative disease? Curr Neurol Neurosci Rep. 2001;1(4):303–305. 11898534 10.1007/s11910-001-0081-z

[110] McGeer PL, McGeer EG. Local neuroinflammation and the progression of Alzheimer’s disease. J Neurovirol. 2002;8(6):529–538. 12476347 10.1080/13550280290100969

[111] Rao SD, Yin HZ, Weiss JH. Disruption of glial glutamate transport by reactive oxygen species produced in motor neurons. J Neurosci. 2003;23(7):2627–2633. 12684448 10.1523/JNEUROSCI.23-07-02627.2003 PMC6742077

[112] Liddelow SA, Guttenplan KA, Clarke LE, et al Neurotoxic reactive astrocytes are induced by activated microglia. Nature. 2017;541(7638):481–487. 28099414 10.1038/nature21029 PMC5404890

[113] Chiu IM, Morimoto ET, Goodarzi H, et al A neurodegeneration-specific gene-expression signature of acutely isolated microglia from an amyotrophic lateral sclerosis mouse model. Cell Rep. 2013;4(2):385–401. 23850290 10.1016/j.celrep.2013.06.018 PMC4272581

[114] Pehar M, Harlan BA, Killoy KM, Vargas MR. Nicotinamide adenine dinucleotide metabolism and neurodegeneration. Antioxidants Redox Signal. 2018;28(18):1652–1668. 10.1089/ars.2017.7145 PMC5962335 28548540

[115] Balbi M, Bonanno G, Bonifacino T, Milanese M. The physio-pathological role of group I metabotropic glutamate receptors expressed by microglia in health and disease with a focus on amyotrophic lateral sclerosis. Int J Mol Sci. 2023;24(6):5240. 36982315 10.3390/ijms24065240 PMC10048889

[116] Raiteri L, Paolucci E, Prisco S, Raiteri M, Bonanno G. Activation of a glycine transporter on spinal cord neurons causes enhanced glutamate release in a mouse model of amyotrophic lateral sclerosis. Br J Pharmacol. 2003;138(6):1021–1025. 12684256 10.1038/sj.bjp.0705142 PMC1573752

[117] Satarker S, Bojja SL, Gurram PC, Mudgal J, Arora D, Nampoothiri M. Astrocytic glutamatergic transmission and its implications in neurodegenerative disorders. Cells. 2022;11(7):1139. 35406702 10.3390/cells11071139 PMC8997779

[118] Pál B . Involvement of extrasynaptic glutamate in physiological and pathophysiological changes of neuronal excitability. Cell Mol Life Sci. 2018;75(16):2917–2949. 29766217 10.1007/s00018-018-2837-5 PMC11105518

[119] Takeuchi H, Suzumura A. Gap junctions and hemichannels composed of connexins: Potential therapeutic targets for neurodegenerative diseases. Front Cell Neurosci. 2014;8:189. 25228858 10.3389/fncel.2014.00189 PMC4151093

[120] Niida-Kawaguchi M, Kakita A, Noguchi N, et al Soluble iron accumulation induces microglial glutamate release in the spinal cord of sporadic amyotrophic lateral sclerosis. Neuropathology. 2020;40(2):152–166. 31883180 10.1111/neup.12632

[121] Arundine M, Tymianski M. Molecular mechanisms of calcium-dependent neurodegeneration in excitotoxicity. Cell Calcium. 2003;34(4-5):325–337. 12909079 10.1016/s0143-4160(03)00141-6

[122] Choi DW . Glutamate neurotoxicity and diseases of the nervous system. Neuron. 1988;1(8):623–634. 2908446 10.1016/0896-6273(88)90162-6

[123] Coyle JT, Puttfarcken P. Oxidative stress, glutamate, and neurodegenerative disorders. Science. 1993;262(5134):689–695. 7901908 10.1126/science.7901908

[124] Tapia R, Medina-Ceja L, Peña F. On the relationship between extracellular glutamate, hyperexcitation and neurodegeneration, in vivo. Neurochem Int. 1999;34(1):23–31. 10100193 10.1016/s0197-0186(98)00061-8

[125] Arundine M, Tymianski M. Molecular mechanisms of glutamate-dependent neurodegeneration in ischemia and traumatic brain injury. Cell Mol Life Sci. 2004;61(6):657–668. 15052409 10.1007/s00018-003-3319-x PMC11138528

[126] McEntee WJ, Crook TH. Glutamate: Its role in learning, memory, and the aging brain. Psychopharmacology (Berl). 1993;111(4):391–401. 7870979 10.1007/BF02253527

[127] Peng S, Zhang Y, Zhang J, Wang H, Ren B. Glutamate receptors and signal transduction in learning and memory. Mol Biol Rep. 2011;38(1):453–460. 20364330 10.1007/s11033-010-0128-9

[128] Weiler IJ, Hawrylak N, Greenough WT. Morphogenesis in memory formation: Synaptic and cellular mechanisms. Behav Brain Res. 1995;66(1-2):1–6. 10.1016/0166-4328(94)00116-w 7755880

[129] Grosskreutz J, Van Den Bosch L, Keller BU. Calcium dysregulation in amyotrophic lateral sclerosis. Cell Calcium. 2010;47(2):165–174. 20116097 10.1016/j.ceca.2009.12.002

[130] Bettler B, Mulle C. AMPA and kainate receptors. Neuropharmacology. 1995;34(2):123–139. 7542368 10.1016/0028-3908(94)00141-e

[131] Hollmann M, Maron C, Heinemann S. N-glycosylation site tagging suggests a three transmembrane domain topology for the glutamate receptor GluR1. Neuron. 1994;13(6):1331–1343. 7993626 10.1016/0896-6273(94)90419-7

[132] Ayala GX, Tapia R. Expression of heat shock protein 70 induced by 4-aminopyridine through glutamate-mediated excitotoxic stress in rat hippocampus in vivo. Neuropharmacology. 2003;45(5):649–660. 12941378 10.1016/s0028-3908(03)00230-2

[133] Ayala GX, Tapia R. Late *N*-methyl-D-aspartate receptor blockade rescues hippocampal neurons from excitotoxic stress and death after 4-aminopyridine-induced epilepsy. Eur J Neurosci. 2005;22(12):3067–3076. 16367773 10.1111/j.1460-9568.2005.04509.x

[134] Peña F, Tapia R. Seizures and neurodegeneration induced by 4-aminopyridine in rat hippocampus in vivo: Role of glutamate- and GABA-mediated neurotransmission and of ion channels. Neuroscience. 2000;101(3):547–561. 11113304 10.1016/s0306-4522(00)00400-0

[135] Burnashev N, Monyer H, Seeburg PH, Sakmann B. Divalent ion permeability of AMPA receptor channels is dominated by the edited form of a single subunit. Neuron. 1992;8(1):189–198. 1370372 10.1016/0896-6273(92)90120-3

[136] Van Damme P, Braeken D, Callewaert G, Robberecht W, Van Den Bosch L. Glur2 deficiency accelerates motor neuron degeneration in a mouse model of amyotrophic lateral sclerosis. J Neuropathol Exp Neurol. 2005;64(7):605–612. 16042312 10.1097/01.jnen.0000171647.09589.07

[137] Laslo P, Lipski J, Nicholson LF, Miles GB, Funk GD. Glur2 AMPA receptor subunit expression in motoneurons at low and high risk for degeneration in amyotrophic lateral sclerosis. Exp Neurol. 2001;169(2):461–471. 11358459 10.1006/exnr.2001.7653

[138] Williams TL, Day NC, Ince PG, Kamboj RK, Shaw PJ. Calcium-permeable α-amino-3-hydroxy-5-methyl-4-isoxazole propionic acid receptors: A molecular determinant of selective vulnerability in amyotrophic lateral sclerosis. Ann Neurol. 1997;42(2):200–207. 9266730 10.1002/ana.410420211

[139] Van Damme P, Callewaert G, Eggermont J, Robberecht W, Van Den Bosch L. Chloride influx aggravates Ca2+-dependent AMPA receptor-mediated motoneuron death. J Neurosci. 2003;23(12):4942–4950. 12832516 10.1523/JNEUROSCI.23-12-04942.2003 PMC6741175

[140] Polster BM, Fiskum G. Mitochondrial mechanisms of neural cell apoptosis. J Neurochem. 2004;90(6):1281–1289. 15341512 10.1111/j.1471-4159.2004.02572.x

[141] Gurney ME, Cutting FB, Zhai P, et al Benefit of vitamin E, riluzole, and gabapeptin in a transgenic model of familial amyotropfic lateral sclerosis. Ann Neurol. 1996;39(2):147–157. 8967745 10.1002/ana.410390203

[142] Roy J, Minotti S, Dong L, Figlewicz DA, Durham HD. Glutamate potentiates the toxicity of mutant Cu/Zn-superoxide dismutase in motor neurons by postsynaptic calcium-dependent mechanisms. J Neurosci. 1998;18(23):9673–9684. 9822728 10.1523/JNEUROSCI.18-23-09673.1998 PMC6793286

[143] Battaglia G, Bruno V. Metabotropic glutamate receptor involvement in the pathophysiology of amyotrophic lateral sclerosis: New potential drug targets for therapeutic applications. Curr Opin Pharmacol. 2018;38:65–71. 29529498 10.1016/j.coph.2018.02.007

[144] Giribaldi F, Milanese M, Bonifacino T, et al Group I metabotropic glutamate autoreceptors induce abnormal glutamate exocytosis in a mouse model of amyotrophic lateral sclerosis. Neuropharmacology. 2013;66:253–263. 22634363 10.1016/j.neuropharm.2012.05.018

[145] Milanese M, Giribaldi F, Melone M, et al Knocking down metabotropic glutamate receptor 1 improves survival and disease progression in the SOD1G93A mouse model of amyotrophic lateral sclerosis. Neurobiol Dis. 2014;64:48–59. 24361555 10.1016/j.nbd.2013.11.006

[146] Bonifacino T, Provenzano F, Gallia E, et al In-vivo genetic ablation of metabotropic glutamate receptor type 5 slows down disease progression in the SOD1G93A mouse model of amyotrophic lateral sclerosis. Neurobiol Dis. 2019;129: 79–92. 31102766 10.1016/j.nbd.2019.05.007

[147] Milanese M, Bonifacino T, Torazza C, et al Blocking glutamate mGlu5 receptors with the negative allosteric modulator CTEP improves disease course in SOD1G93A mouse model of amyotrophic lateral sclerosis. Br J Pharmacol. 2021;178(18):3747–3764. 33931856 10.1111/bph.15515 PMC8457068

[148] Krieger C, Jones K, Kim SU, Eisen AA. The role of intracellular free calcium in motor neuron disease. J Neurol Sci. 1994;124:27–32. 7807138 10.1016/0022-510x(94)90173-2

[149] Andressen C, Blümcke I, Celio MR. Calcium-binding proteins: Selective markers of nerve cells. Cell Tissue Res. 1993;271(2):181–208. 8453652 10.1007/BF00318606

[150] Baimbridge KG, Celio MR, Rogers JH. Calcium-binding proteins in the nervous system. Trends Neurosci. 1992;15(8):303–308. 1384200 10.1016/0166-2236(92)90081-i

[151] de la Cruz RR, Pastor AM, Martínez-Guijarro FJ, López-García C, Delgado-García JM. Localization of parvalbumin, calretinin, and calbindin D-28k in identified extraocular motoneurons and internuclear neurons of the cat. J Comp Neurol. 1998;390(3):377–391. 9455899 10.1002/(sici)1096-9861(19980119)390:3<377::aid-cne6>3.0.co;2-z

[152] Ince P, Stout N, Shaw P, et al Parvalbumin and calbindin D-28k in the human motor system and in motor neuron disease. Neuropathol Appl Neurobiol. 1993;19(4):291–299. 8232749 10.1111/j.1365-2990.1993.tb00443.x

[153] Beers DR, Ho BK, Siklós L, et al Parvalbumin overexpression alters immune-mediated increases in intracellular calcium, and delays disease onset in a transgenic model of familial amyotrophic lateral sclerosis. J Neurochem. 2001;79(3):499–509. 11701753 10.1046/j.1471-4159.2001.00582.x

[154] Van Den Bosch L, Schwaller B, Vleminckx V, et al Protective effect of parvalbumin on excitotoxic motor neuron death. Exp Neurol. 2002;174(2):150–161. 11922657 10.1006/exnr.2001.7858

[155] Laslo P, Lipski J, Nicholson LF, Miles GB, Funk GD. Calcium binding proteins in motoneurons at low and high risk for degeneration in ALS. Neuroreport. 2000;11(15):3305–3308. 11059892 10.1097/00001756-200010200-00009

[156] Grosskreutz J, Haastert K, Dewil M, et al Role of mitochondria in kainate-induced fast Ca^2+^ transients in cultured spinal motor neurons. Cell Calcium. 2007;42(1):59–69. 17241659 10.1016/j.ceca.2006.11.010

[157] Alexianu ME, Ho B, Mohamed AH, La Bella V, Smith RG, Appel SH. The role of calcium-binding proteins in selective motoneuron vulnerability in amyotrophic lateral sclerosis. Ann Neurol. 1994;36(6):846–858. 7998770 10.1002/ana.410360608

[158] Palecek J, Lips MB, Keller BU, Physiologie Z, Universit G. Calcium dynamics and buffering in motoneurones of the mouse spinal cord. J Physiol. 1999;520(2):485–502. 10523417 10.1111/j.1469-7793.1999.00485.x PMC2269591

[159] Reiner A, Medina L, Figueredo-Cardenas G, Anfinson S. Brainstem motoneuron pools that are selectively resistant in amyotrophic lateral sclerosis are preferentially enriched in parvalbumin: Evidence from monkey brainstem for a calcium-mediated mechanism in sporadic ALS. Exp Neurol. 1995;131(2):239–250. 7895823 10.1016/0014-4886(95)90046-2

[160] Siklös L, Engelhardt JI, Alexianu ME, Gurney ME, Siddique T, Appel SH. Intracellular calcium parallels motoneuron degeneration in SOD-1 mutant mice. J Neuropathol Exp Neurol. 1998;57(6):571–587. 9630237 10.1097/00005072-199806000-00005

[161] Dekkers J, Bayley P, Dick JRT, Schwaller B, Berchtold MW, Greensmith L. Over-expression of parvalbumin in transgenic mice rescues motoneurons from injury-induced cell death. Neuroscience. 2004;123(2):459–466. 14698753 10.1016/j.neuroscience.2003.07.013

[162] Philippe E, Gaulin F, Audet G, Zhou C. Expression of gamma-aminobutyric acid and calcium binding protein-parvalbumin by chick motoneurons. Brain Res Bull. 1993;30(3-4):325–328. 8457881 10.1016/0361-9230(93)90260-i

[163] Bouilleret V, Schwaller B, Schurmans S, Celio MR, Fritschy JM. Neurodegenerative and morphogenic changes in a mouse model of temporal lobe epilepsy do not depend on the expression of the calcium-binding proteins parvalbumin, calbindin, or calretinin. Neuroscience. 2000;97(1):47–58. 10771338 10.1016/s0306-4522(00)00017-8

[164] Shaw PJ, Eggett CJ. Molecular factors underlying selective vulnerability of motor neurons to neurodegeneration in amyotrophic lateral sclerosis. J Neurol. 2000;247:I17–I27. 10795883 10.1007/BF03161151

[165] Lips MB, Keller BU. Endogenous calcium buffering in motoneurones of the nucleus hypoglossus from mouse. J Physiol. 1998;511(1):105–117. 9679167 10.1111/j.1469-7793.1998.105bi.x PMC2231095

[166] Vanselow BK, Keller BU. Calcium dynamics and buffering in oculomotor neurones from mouse that are particularly resistant during amyotrophic lateral sclerosis (ALS)-related motoneurone disease. J Physiol. 2000;525(Pt 2):433–445. 10835045 10.1111/j.1469-7793.2000.t01-1-00433.x PMC2269959

[167] Résibois A, Rogers JH. Calretinin in rat brain: An immunohistochemical study. Neuroscience. 1992;46(1):101–134. 1594096 10.1016/0306-4522(92)90012-q

[168] Chard PS, Bleakman D, Christakos S, Fullmer CS, Miller RJ. Calcium buffering properties of calbindin D28k and parvalbumin in rat sensory neurones. J Physiol. 1993;472(1):341–357. 8145149 10.1113/jphysiol.1993.sp019950 PMC1160490

[169] Lledo PM, Homburger V, Bockaert J, Vincent JD. Differential G protein-mediated coupling of D2 dopamine receptors to K+ and Ca^2+^ currents in rat anterior pituitary cells. Neuron. 1992;8(3):455–463. 1312848 10.1016/0896-6273(92)90273-g

[170] Mattson MP, Rychlik B, Chu C, Christakost S. Evidence for calcium-reducing and excitoprotective roles for the calcium-binding protein calbindin-1328k in cultured hippocampal neurons. Neuron. 1991;6(1):41–51. 1670921 10.1016/0896-6273(91)90120-o

[171] Agrawal I, Lim YS, Ng SY, Ling SC. Deciphering lipid dysregulation in ALS: From mechanisms to translational medicine. Transl Neurodegener. 2022;11(1):48. 36345044 10.1186/s40035-022-00322-0 PMC9641964

[172] Ho WY, Hartmann H, Ling SC. Central nervous system cholesterol metabolism in health and disease. IUBMB Life. 2022;74:826–841. 35836360 10.1002/iub.2662

[173] Bernal AF, Mota N, Pamplona R, Area-Gomez E, Portero-Otin M. Hakuna MAM-Tata: Investigating the role of mitochondrial-associated membranes in ALS. Biochim Biophys Acta Mol Basis Dis. 2023;1869(6):166716. 37044239 10.1016/j.bbadis.2023.166716

[174] von Lewinski F, Keller BU. Ca2+, mitochondria and selective motoneuron vulnerability: Implications for ALS. Trends Neurosci. 2005;28(9):494–500. 16026864 10.1016/j.tins.2005.07.001

[175] Andreassen OA, Dedeoglu A, Ferrante RJ, et al Creatine increases survival and delays motor symptoms in a transgenic animal model of Huntington’s disease. Neurobiol Dis. 2001;8(3):479–491. 11447996 10.1006/nbdi.2001.0406

[176] Kruman II, Pedersen WA, Springer JE, Mattson MP. ALS-linked Cu/Zn-SOD mutation increases vulnerability of motor neurons to excitotoxicity by a mechanism involving increased oxidative stress and perturbed calcium homeostasis. Exp Neurol. 1999;160(1):28–39. 10630188 10.1006/exnr.1999.7190

[177] Rao SD, Weiss JH. Excitotoxic and oxidative cross-talk between motor neurons and glia in ALS pathogenesis. Trends Neurosci. 2004;27(1):17–23. 14698606 10.1016/j.tins.2003.11.001

[178] Swerdlow RH, Parks JK, Cassarino DS, et al Mitochondria in sporadic amyotrophic lateral sclerosis. Exp Neurol. 1998;153(1):135–142. 9743575 10.1006/exnr.1998.6866

[179] Mathis S, Goizet C, Soulages A, Vallat JM, Le Masson G. Genetics of amyotrophic lateral sclerosis: A review. J Neurol Sci. 2019;399(February):217–226. 30870681 10.1016/j.jns.2019.02.030

[180] Granatiero V, Manfredi G. Mitochondrial transport and turnover in the pathogenesis of amyotrophic lateral sclerosis. Biology (Basel). 2019;8(2):36. 31083575 10.3390/biology8020036 PMC6627920

[181] Wang H, Kodavati M, Britz GW, Hegde ML. DNA damage and repair deficiency in ALS/FTD-associated neurodegeneration: From molecular mechanisms to therapeutic implication. Front Mol Neurosci. 2021;14:784361. 34975400 10.3389/fnmol.2021.784361 PMC8716463

[182] Mejzini R, Flynn LL, Pitout IL, Fletcher S, Wilton SD, Akkari PA. ALS genetics, mechanisms, and therapeutics: Where are we now? Front Neurosci. 2019;13:1310. 31866818 10.3389/fnins.2019.01310 PMC6909825

[183] Dasuri K, Zhang L, Keller JN. Oxidative stress, neurodegeneration, and the balance of protein degradation and protein synthesis. Free Radic Biol Med. 2013;62:170–185. 23000246 10.1016/j.freeradbiomed.2012.09.016

[184] Valko M, Leibfritz D, Moncol J, Cronin MTD, Mazur M, Telser J. Free radicals and antioxidants in normal physiological functions and human disease. Int J Biochem Cell Biol. 2007;39(1):44–84. 16978905 10.1016/j.biocel.2006.07.001

[185] Halliwell B . Free radicals and antioxidants: Updating a personal view. Nutr Rev. 2012;70(5):257–265. 22537212 10.1111/j.1753-4887.2012.00476.x

[186] Prasad M, Kaur J, Pawlak KJ, Bose M, Whittal RM, Bose HS. Mitochondria-associated endoplasmic reticulum membrane (MAM) regulates steroidogenic activity via steroidogenic acute regulatory protein (StAR)-voltage-dependent anion channel 2 (VDAC2) interaction. J Biol Chem. 2015;290(5):2604–2616. 25505173 10.1074/jbc.M114.605808 PMC4317014

[187] Cassina P, Cassina A, Pehar M, et al Mitochondrial dysfunction in SOD1G93A-bearing astrocytes promotes motor neuron degeneration: Prevention by mitochondrial-targeted antioxidants. J Neurosci. 2008;28(16):4115–4122. 18417691 10.1523/JNEUROSCI.5308-07.2008 PMC3844766

[188] Ilieva H, Polymenidou M, Cleveland DW. Non-cell autonomous toxicity in neurodegenerative disorders: ALS and beyond. J Cell Biol. 2009;187(6):761–772. 19951898 10.1083/jcb.200908164 PMC2806318

[189] Radi R . Protein tyrosine nitration: Biochemical mechanisms and structural basis of functional effects. Acc Chem Res. 2013;46(2):550–559. 23157446 10.1021/ar300234c PMC3577981

[190] Barbeito LH, Pehar M, Cassina P, et al A role for astrocytes in motor neuron loss in amyotrophic lateral sclerosis. Brain Res Rev. 2004;47(1-3):263–274. 15572176 10.1016/j.brainresrev.2004.05.003

[191] Trotti D, Rossi D, Gjesdal O, et al Peroxynitrite inhibits glutamate transporter subtypes. J Biol Chem. 1996;271(11):5976–5979. 8626378 10.1074/jbc.271.11.5976

[192] Kerr JFR, Wyllie AH, Currie AR. Apoptosis: A basic biological phenomenon with wide-ranging implications in tissue kinetics. Br J Cancer. 1972;26(4):239–257. 4561027 10.1038/bjc.1972.33 PMC2008650

[193] Gorman AM . Neuronal cell death in neurodegenerative diseases: Recurring themes around protein handling: Apoptosis Review Series. J Cell Mol Med. 2008;12(6a):2263–2280. 18624755 10.1111/j.1582-4934.2008.00402.x PMC4514105

[194] Carlsson SK, Brothers SP, Wahlestedt C. Emerging treatment strategies for glioblastoma multiforme. EMBO Mol Med. 2014;6(11):1359–1370. 25312641 10.15252/emmm.201302627 PMC4237465

[195] Moujalled D, Strasser A, Liddell JR. Molecular mechanisms of cell death in neurological diseases. Cell Death Differ. 2021;28(7):2029–2044. 34099897 10.1038/s41418-021-00814-y PMC8257776

[196] Dong XX, Wang Y, Qin ZH. Molecular mechanisms of excitotoxicity and their relevance to pathogenesis of neurodegenerative diseases. Acta Pharmacol Sin. 2009;30(4):379–387. 19343058 10.1038/aps.2009.24 PMC4002277

[197] Soto C, Pritzkow S. Protein misfolding, aggregation, and conformational strains in neurodegenerative diseases. Nat Neurosci. 2018;21(10):1332–1340. 30250260 10.1038/s41593-018-0235-9 PMC6432913

[198] Zhan L, Xie Q, Tibbetts RS. Opposing roles of p38 and JNK in a Drosophila model of TDP-43 proteinopathy reveal oxidative stress and innate immunity as pathogenic components of neurodegeneration. Hum Mol Genet. 2015;24(3):757–772. 25281658 10.1093/hmg/ddu493 PMC4291250

[199] Prasad A, Bharathi V, Sivalingam V, Girdhar A, Patel BK. Molecular mechanisms of TDP-43 misfolding and pathology in amyotrophic lateral sclerosis. Front Mol Neurosci. 2019;12:25. 30837838 10.3389/fnmol.2019.00025 PMC6382748

[200] Aikio M, Odeh HM, Wobst HJ, et al Opposing roles of p38α-mediated phosphorylation and PRMT1-mediated arginine methylation in driving TDP-43 proteinopathy. Cell Rep. 2025;44(1):115205. 39817908 10.1016/j.celrep.2024.115205 PMC11831926

[201] Bradley WG, Anderson F, Bromberg M, et al Current management of ALS: Comparison of the ALS CARE Database and the AAN Practice Parameter. Neurology. 2001;57(3):500–504. 11502920 10.1212/wnl.57.3.500

[202] Van Den Berg JP, Kalmijn S, Lindeman E, et al Multidisciplinary ALS care improves quality of life in patients with ALS. Neurology. 2005;65(8):1264–1267. 16247055 10.1212/01.wnl.0000180717.29273.12

[203] Traynor BJ, Alexander M, Corr B, Frost E, Hardiman O. Effect of a multidisciplinary amyotrophic lateral sclerosis (ALS) clinic on ALS survival: A population based study, 1996–2000. J Neurol Neurosurg Psychiatry. 2003;74(9):1258–1261. 12933930 10.1136/jnnp.74.9.1258 PMC1738639

[204] Miller RG, Anderson F, Brooks BR, Mitsumoto H, Bradley WG, Ringel SP. Outcomes research in amyotrophic lateral sclerosis: Lessons learned from the amyotrophic lateral sclerosis clinical assessment, research, and education database. Ann Neurol. 2009;65(Suppl. 1):S24–S28. 19191307 10.1002/ana.21556

[205] Tzeplaeff L, Wilfling S, Requardt MV, Herdick M. Current state and future directions in the therapy of ALS. Cells. 2023;12(11):1523. 37296644 10.3390/cells12111523 PMC10252394

[206] Hinchcliffe M, Smith A. Riluzole: Real-world evidence supports significant extension of median survival times in patients with amyotrophic lateral sclerosis. Degener Neurol Neuromuscul Dis. 2017;7:61–70. 30050378 10.2147/DNND.S135748 PMC6053101

[207] Amado DA, Davidson BL. Gene therapy for ALS: A review. Mol Ther. 2021;29(12):3345–3358. 33839324 10.1016/j.ymthe.2021.04.008 PMC8636154

[208] Bensimon G, Lacomblez L, Meininger V. A controlled trial of riluzole in amyotrophic lateral sclerosis. N Engl J Med. 1994;330(9):585–591. 8302340 10.1056/NEJM199403033300901

[209] Vasta R, Ombelet F, Hobin F, et al Real-world prognostic role of riluzole use in ALS: A multi-center study from PRECISION-ALS. Amyotroph Lateral Scler Front Degener. 2025;26(suppl 1):50–60. 10.1080/21678421.2025.2472889 40326914

[210] Chiò A, Mazzini L, Mora G. Disease-modifying therapies in amyotrophic lateral sclerosis. Neuropharmacology. 2020;167:107986. 32062193 10.1016/j.neuropharm.2020.107986

[211] Foran E, Trotti D. Glutamate transporters and the excitotoxic path to motor neuron degeneration in amyotrophic lateral sclerosis. Antioxidants Redox Signal. 2009;11(7):1587–1602. 10.1089/ars.2009.2444 PMC2842587 19413484

[212] Rothstein JD, Patel S, Regan MR, et al β-Lactam antibiotics offer neuroprotection by increasing glutamate transporter expression. Nature. 2005;433(7021):73–77. 15635412 10.1038/nature03180

[213] Turalde CWR, Moalong KMC, Espiritu AI, Prado MB. Perampanel for amyotrophic lateral sclerosis: A systematic review and meta-analysis. Neurol Sci. 2022;43(2):889–897. 34994876 10.1007/s10072-022-05867-6

[214] Patai R, Paizs M, Tortarolo M, et al Presymptomatically applied AMPA receptor antagonist prevents calcium increase in vulnerable type of motor axon terminals of mice modeling amyotrophic lateral sclerosis. Biochim Biophys Acta Mol Basis Dis. 2017;1863(7):1739–1748. 28528135 10.1016/j.bbadis.2017.05.016

[215] Paizs M, Tortarolo M, Bendotti C, Engelhardt JI, Siklós L. Talampanel reduces the level of motoneuronal calcium in transgenic mutant SOD1 mice only if applied presymptomatically. Amyotroph Lateral Scler. 2011;12(5):340–344. 21623665 10.3109/17482968.2011.584627 PMC3231880

[216] Pascuzzi RM, Shefner J, Chappell AS, et al A phase II trial of talampanel in subjects with amyotrophic lateral sclerosis. Amyotroph Lateral Scler. 2010;11(3):266–271. 19961264 10.3109/17482960903307805

[217] Wang R, Zhang D. Memantine prolongs survival in an amyotrophic lateral sclerosis mouse model. Eur J Neurosci. 2005;22(9):2376–2380. 16262676 10.1111/j.1460-9568.2005.04431.x

[218] Bhai S, Bowser R, Moser S, et al A 40-week phase 2B randomized, multicenter, double-blind, placebo-controlled study evaluating the safety and efficacy of memantine in amyotrophic lateral sclerosis (P8-8.011). Neurology. 2023;100(17_supplement_2):4795. 10.1002/mus.28287 PMC11632565 39511965

[219] Spittel S, Grehl T, Weydt P, et al Dextromethorphan/quinidine (DMQ) for reducing bulbar symptoms in amyotrophic lateral sclerosis–assessment of treatment experience in a multicenter study. Amyotroph Lateral Scler Front Degener. 2026;27(1-2):185–197. 10.1080/21678421.2025.2557932 40932199

[220] Benatar M, Wuu J, Andersen PM, et al Design of a randomized, placebo-controlled, phase 3 trial of tofersen initiated in clinically presymptomatic SOD1 variant carriers: The ATLAS study. Neurotherapeutics. 2022;19(4):1248–1258. 35585374 10.1007/s13311-022-01237-4 PMC9587202

[221] Miller TM, Cudkowicz ME, Genge A, et al Trial of antisense oligonucleotide tofersen for *SOD1* ALS. N Engl J Med. 2022;387(12):1099–1110. 36129998 10.1056/NEJMoa2204705

[222] Benatar M, Wuu J. Presymptomatic studies in ALS: Rationale, challenges, and approach. Neurology. 2012;79(16):1732–1739. 23071166 10.1212/WNL.0b013e31826e9b1d PMC3468777

[223] Blair HA . Tofersen: First approval. Drugs. 2023;83(11):1039–1043. 37316681 10.1007/s40265-023-01904-6

[224] Asundi A, Cervantes-Arslanian AM, Lin NH, Barbosa F. Infectious myelitis. Semin Neurol. 2019;39(4):472–481. 31533188 10.1055/s-0039-1688923

[225] Reilich P, Schöberl F, Hiebeler M, Tonon M, Ludolph AC, Senel M. Myelitis as a side effect of tofersen therapy in SOD1-associated ALS. J Neurol. 2024;271(4):2114–2118. 38066205 10.1007/s00415-023-12130-1 PMC10973064

[226] Sparasci D, Castelli C, Staedler C, Gobbi C, Ripellino P. Inclusions in macrophages of the cerebrospinal fluid during treatment with Tofersen. Muscle Nerve. 2023;67(2):E3–E5. 36477882 10.1002/mus.27763

[227] European Medicines Agency (EMA). EMA product number EMEA/H/C/005493. Qalsody; 2024. https://www.ema.europa.eu/en/medicines/human/EPAR/qalsody

[228] Sreedharan J, Brown RH. Amyotrophic lateral sclerosis: Problems and prospects. Ann Neurol. 2013;74(3):309–316. 24038380 10.1002/ana.24012

[229] Fujimori K, Ishikawa M, Otomo A, et al Modeling sporadic ALS in iPSC-derived motor neurons identifies a potential therapeutic agent. Nat Med. 2018;24(10):1579–1589. 30127392 10.1038/s41591-018-0140-5

[230] Hoye ML, Regan MR, Jensen LA, et al Motor neuron-derived microRNAs cause astrocyte dysfunction in amyotrophic lateral sclerosis. Brain. 2018;141(9):2561–2575. 30007309 10.1093/brain/awy182 PMC6113638

[231] Rothstein JD, Dykes-Hoberg M, Pardo CA, et al Knockout of glutamate transporters reveals a major role for astroglial transport in excitotoxicity and clearance of glutamate. Neuron. 1996;16(3):675–686. 8785064 10.1016/s0896-6273(00)80086-0

